# Chemical genetic screen identifies lithocholic acid as an anti-aging compound that extends yeast chronological life span in a TOR-independent manner, by modulating housekeeping longevity assurance processes

**DOI:** 10.18632/aging.100168

**Published:** 2010-07-07

**Authors:** Alexander A. Goldberg, Vincent R. Richard, Pavlo Kyryakov, Simon D. Bourque, Adam Beach, Michelle T. Burstein, Anastasia Glebov, Olivia Koupaki, Tatiana Boukh-Viner, Christopher Gregg, Mylène Juneau, Ann M. English, David Y. Thomas, Vladimir I. Titorenko

**Affiliations:** ^1^ Department of Biology , Concordia University, Montreal, Quebec H4B 1R6, Canada; ^2^ Department of Chemistry and Biochemistry, Concordia University, Montreal, Quebec H4B 1R6, Canada; ^3^ Department of Biochemistry, McGill University, Montreal, Quebec H3G 1Y6, Canada; ^4^ These authors contributed equally to this work

**Keywords:** Cellular aging, longevity, yeast, caloric restriction, chemical biology, anti-aging compounds

## Abstract

In chronologically aging yeast, longevity can be extended by administering a caloric restriction (CR) diet or some small molecules. These life-extending interventions target the adaptable target of rapamycin (TOR) and cAMP/protein kinase A (cAMP/PKA) signaling pathways that are under the stringent control of calorie availability. We designed a chemical genetic screen for small molecules that increase the chronological life span of yeast under CR by targeting lipid metabolism and modulating housekeeping longevity pathways that regulate longevity irrespective of the number of available calories. Our screen identifies lithocholic acid (LCA) as one of such molecules. We reveal two mechanisms underlying the life-extending effect of LCA in chronologically aging yeast. One mechanism operates in a calorie availability-independent fashion and involves the LCA-governed modulation of housekeeping longevity assurance pathways that do not overlap with the adaptable TOR and cAMP/PKA pathways. The other mechanism extends yeast longevity under non-CR conditions and consists in LCA-driven unmasking of the previously unknown anti-aging potential of PKA. We provide evidence that LCA modulates housekeeping longevity assurance pathways by suppressing lipid-induced necrosis, attenuating mitochondrial fragmentation, altering oxidation-reduction processes in mitochondria, enhancing resistance to oxidative and thermal stresses, suppressing mitochondria-controlled apoptosis, and enhancing stability of nuclear and mitochondrial DNA.

## Introduction

Aging of multicellular and unicellular eukaryotic organisms is a multifactorial biological phenomenon that has various causes and affects a plethora of cellular activities [1]. These numerous activities are modulated by only a few nutrient- and energy-sensing signaling pathways that are conserved across phyla and include the insulin/insulin-like growth factor 1 (IGF-1), AMP-activated protein  kinase/target of rapamycin  (AMPK/ TOR) and cAMP/protein kinase A (cAMP/PKA) pathways [2-5]. By sharing a compendium of protein kinases and adaptor proteins, the insulin/IGF-1, AMPK/TOR and cAMP/PKA pathways in yeast, worms, fruit flies and mammals converge into a network regulating longevity [2-4],^6^,^7^]. This network may also include several proteins that currently are not viewed as being in any of these three pathways [2,3,8,9]. Moreover, this network responds to the age-related partial mitochondrial dysfunction and is modulated by mitochondrially produced reactive oxygen species (ROS) [3,8,10,11]. By sensing the nutritional status of the whole organism as well as the intracellular nutrient and energy status, functional state of mitochondria, and concentration of ROS produced in mitochondria, the longevity network regulates life span across species by coordinating information flow along its convergent, divergent and multiply branched signaling pathways.

By defining the organismal and intracellular nutrient and energy status, nutrient intake plays an important role in modulating life span and influences age-related pathologies [12,13]. Two dietary regimens are known to have the most profound life-extending effects across species and to improve overall health by delaying the onset of age-related diseases. They include: 1) caloric restriction (CR), a diet in which only calorie intake is reduced but the supply of amino acids, vitamins and other nutrients is not compromised [13-15]; and 2) dietary restriction (DR), in which the intake of nutrients (but not necessarily of calories) is reduced by limiting food supply without causing malnutrition [16-18]. In a "TOR-centric" view of longevity regulation, TOR alone governs the life-extending and health-improving effects of CR/DR by: 1) integrating the flow of information on the organismal and intracellular nutrient and energy status from the protein kinases AMPK, PKA, PKB/AKT (the insulin/IGF-1 pathway) and ERK1/2 (the PKA-inhibited Raf/MEK/ERK protein kinase cascade) as well as from the mitochondrial redox protein P66^Shc^; 2) sensing the intracellular levels of amino acids in an AMPK-independent manner; and 3) operating as a control center which, based on the information it has gathered and processed, modulates many longevity-related processes in a sirtuin-independent fashion [19-21]. The inability of CR to increase the replicative life span (RLS) of yeast mutants lacking components of the TOR pathway [22] and the lack of the beneficial effect of DR on life span in worms with reduced TOR signaling [23,24] support the proposed central role for TOR in orchestrating the life-extending effect of CR/DR in these two longevity paradigms. Moreover, while the postulated by the TOR-centric model dispensability of sirtuins for the longevity benefit associated with DR has been confirmed in worms [24], the importance of the sirtuin Sir2p in mediating the life-extending effect of CR in replicatively aging yeast is debated [22,25-[27]. Noteworthy, while TOR is a central regulator of the life-extending effect of CR in replicatively aging yeast, the longevity benefit associated with CR in chronologically aging yeast is mediated by a signaling network that includes: 1) the TOR and cAMP/PKA pathways converged on Rim15p, which therefore acts as a nutritional integrator; and 2) some other, currently unknown pathways that are not centered on Rim15p [6]. Considering the likely convergence of the insulin/IGF-1, AMPK/TOR and cAMP/PKA signaling pathways into a network regulating longevity in worms, fruit flies and mammals (see above), it is conceivable that - akin to TOR - the insulin/IGF-1 and cAMP/PKA pathways may contribute to the beneficial effect of CR/DR on their longevity. Although some findings in worms, fruit flies and mammals support the involvement of the insulin/IGF-1 pathway in the longevity benefit associated with CR/DR, other data imply that such benefit is independent of insulin/IGF-1 (reviewed by Narasimhan *et al*. [3]). The role of cAMP/PKA signaling in the life-extending effect of CR/DR in these multicellular eukaryotes remains to be tested. Importantly, the recently reported in worms involvement of both independent and overlapping pathways in life span extension by different DR regimens [28] supports the notion that the longevity benefit associated with nutrient limitation is mediated by a signaling network that integrates several pathways.

Akin to CR and DR regimens, certain pharmacological interventions can extend longevity across phyla and improve health by beneficially influencing age-related pathologies. Noteworthy, all of the currently known anti-aging compounds increase life span under non-CR or non-DR conditions (Supplementary Table 1). Under such conditions, these compounds have been shown to: 1) provide the longevity and health benefits associated with CR and DR, but without restricting caloric and nutrient intake; and 2) mimic numerous life-extending effects of CR and DR on gene expression pattern, metabolic and physiological processes, and stress response pathways. Therefore, the names "CR mimetics" and "DR mimetics" have been coined for them [29,30]. Importantly, most CR mimetics and DR mimetics target signaling pathways that modulate longevity in response to the organismal and intracellular nutrient and energy status, including the insulin/IGF-1 and AMPK/TOR pathways as well as the sirtuin-governed protein deacetylation module of the longevity signaling network integrating these pathways (Supplementary Table 1). Furthermore, such compounds as resveratrol, metformin and mianserin increase life span only under non-CR or non-DR conditions, but are unable to do so if the supply of calories or nutrients is limited [31-35]. Hence, one could envision that most, if not all, longevity pathways are "adaptable" by nature, *i.e.*, that they modulate longevity only in response to certain changes in the extracellular and intracellular nutrient and energy status of an organism. However, Li^+^ in worms and rapamycin in fruits flies extend life span even under DR conditions [36,37]. It is likely therefore that some longevity pathways could be "constitutive" or "housekeeping" by nature, *i.e.*, that they: 1) modulate longevity irrespective of the organismal and intra-cellular nutrient and energy status; and 2) do not overlap (or only partially overlap) with the adaptable longevity pathways that are under the stringent control of calorie and/or nutrient availability. The challenge is to identify such housekeeping longevity pathways, perhaps by using a chemical screen for compounds that can extend longevity even under CR/DR conditions. Because under such conditions the adaptable pro-aging pathways are fully suppressed and the adaptable anti-aging pathways are fully activated, a compound that can increase life span is expected to target the housekeeping longevity pathways.

Noteworthy, two anti-aging compounds alter lipid levels in mammals and fruit flies under non-DR conditions. In fact, resveratrol treatment reduces the levels of the neutral lipids triacylglycerols (TAG) and increases free fatty acid (FFA) levels in mouse adipocytes [38]. Furthermore, feeding rapamycin to fruit flies results in elevated TAG levels [37]. Although it remains to be seen if such effects of resveratrol and rapamycin on lipid levels play a casual role in their anti-aging action under non-DR conditions, it should be stressed that lipid metabolism has been shown to be involved in longevity regulation in yeast [39,40], worms [9,41-43], fruit flies [41,44] and mice [38,41,45-48]. We recently proposed a mechanism linking yeast longevity and lipid dynamics in the endoplasmic reticulum (ER), lipid bodies and peroxisomes. In this mechanism, a CR diet extends yeast chronological life span (CLS) by activating FFA oxidation in peroxisomes [39-40]. It is conceivable that the identification of small molecules targeting this mechanism could yield novel anti-aging compounds. Such compounds can be used as research tools for defining the roles for different longevity pathways in modulating lipid metabolism and in integrating lipid dynamics with other longevity-related processes. Furthermore, the availability of such compounds would enable a quest for housekeeping longevity assurance pathways that do not overlap (or only partially overlap) with the adaptable TOR and cAMP/PKA pathways. Moreover, such compounds would have a potential to be used as pharmaceutical agents for increasing life span and promoting healthy aging by delaying the onset of age-related diseases, regardless of an organism's dietary regimen.

We sought to identify small molecules that increase the CLS of yeast under CR conditions by targeting lipid metabolism and modulating housekeeping longevity assurance pathways. Our chemical genetic screen identified lithocholic acid (LCA) as one of such small molecules. We provide evidence that LCA extends longevity of chronologically aging yeast through two different mechanisms. In one mechanism, this bile acid targets - regardless of the number of available calories - housekeeping longevity assurance pathways that do not overlap with the adaptable TOR and cAMP/PKA pathways and modulate a compendium of pro- and anti-aging processes. In the other mechanism, LCA targets the adaptable cAMP/PKA pathway under non-CR conditions by unmasking the previously unknown anti-aging potential of PKA.

## Results

### Our rationale for choosing a mutant strain and growth conditions to screen compound libraries for anti-aging small molecules

**Figure 1.**
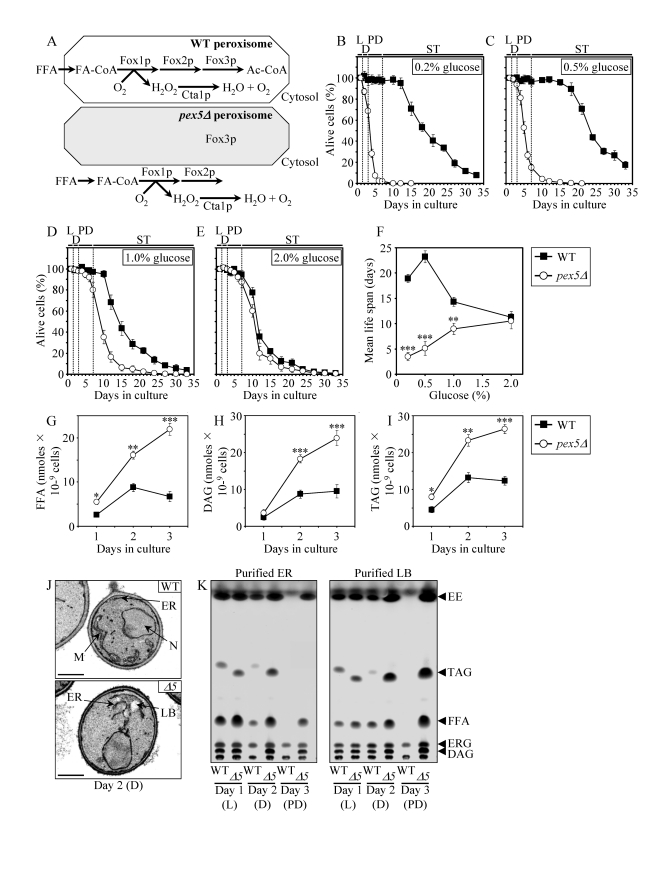
** The *pex5Δ* mutation shortens chronological life span (CLS), alters cell morphology and remodels lipid metabolism in CR yeast.** (**A**) Outline of subcellular localization of the Fox1p, Fox2p and Fox3p enzymes of fatty acid ß-oxidation in WT and *pex5Δ* cells. (**B** - **F**) Survival and the mean life spans of chronologically aging WT and *pex5Δ *yeast cultured in medium initially containing 0.2%, 0.5%, 1% or 2% glucose. Data are presented as means ± SEM (n = 16-38; ***p < 0.001; **p < 0.01). (**G** - **I**) Levels of free fatty acids (FFA), diacylglycerols (DAG) and triacylglycerols (TAG) in WT and *pex5Δ *cells grown on 0.2% glucose and taken for analyses at the indicated time-points. FFA and TAG  were measured by quantitative mass spectrometry. The levels of DAG were quantitated by densitometric analysis of TLC plates. Data are presented as means ± SEM (n = 3-8; ***p < 0.001; **p < 0.01; *p < 0.05). (**J **and **K**) Transmission electron micrographs (**J**) and spectra of lipids extracted from purified endoplasmic reticulum (ER) and lipid bodies (LB) and analyzed by TLC (**K**) for WT and *pex5Δ *(*Δ5*) yeast grown on 0.2% glucose and taken for analyses at the indicated time-points. Abbreviations: Cta1p, peroxisomal catalase; D, diauxic growth phase; EE, ethyl esters; ERG, ergosterol; FA-CoA, CoA esters of fatty acids; L, logarithmic growth phase; M, mitochondrion; N, nucleus; PD, post-diauxic growth phase; ST, stationary growth phase.

To perform a chemical genetic screen for small molecules that increase the CLS of yeast by targeting lipid metabolism, we chose the single-gene-deletion mutant strain *pex5Δ*. Because *pex5Δ* lacks a cytosolic shuttling receptor for peroxisomal import of Fox1p and Fox2p, these two enzymes of the β-oxidation of FFA reside in the cytosol of *pex5Δ* cells [49] (Figure 1A). In contrast, the Pex5p-independent peroxisomal import of Fox3p, the third enzyme of the FFA β-oxidation pathway, sorts it to the peroxisome in *pex5Δ* cells [49]. By spatially separating Fox1p and Fox2p from Fox3p within a cell, the *pex5Δ* mutation impairs FFA oxidation (Figure 1A). In chronologically aging yeast grown under CR conditions on 0.2% or 0.5% glucose, peroxisomal FFA oxidation regulates longevity by 1) efficiently generating acetyl-CoA to synthesize the bulk of ATP in mitochondria; and 2) acting as a rheostat that modulates the age-related dynamics of FFA and diacylglycerol (DAG), two regulatory lipids known to promote longevity-defining cell death [39,40,50]. Unlike CR yeast, chronologically aging non-CR yeast grown on 1% or 2% glucose are unable to generate significant quantities of ATP by oxidizing peroxisome-derived acetyl-CoA in mitochondria and, instead, produce the bulk of ATP via glycolytic oxidation of glycogen- and trehalose-derived glucose [39,40]. Consistent with the essential role of peroxisomal FFA oxidation as a longevity assurance process only under CR, the *pex5Δ* mutation substantially shortened the CLS of CR yeast but caused a significantly lower reduction of longevity in non-CR yeast, especially in yeast grown on 2% glucose (Figures 1B to F).

In chronologically aging CR yeast, peroxisomal FFA oxidation modulates, perhaps via several negative feedback loops, the following three processes: 1) the ER-confined biosynthesis of TAG from FFA and DAG; 2) the subsequent deposition of TAG, the major neutral lipid reserves, in lipid bodies; and 3) the consequent lipolysis of deposited TAG and the resulting formation of FFA and DAG [39,40]. By impairing the ability of peroxisomal FFA oxidation to act as a rheostat that regulates cellular aging by modulating the age-related dynamics of FFA, DAG and TAG in the ER and lipid bodies, the *pex5Δ* mutation caused the accumulation of the closely apposed ER membranes and ER-originated lipid bodies in CR yeast (Figure 1J). Of note, these morphological features of *pex5Δ* yeast under CR were similar to those observed in a mouse model for the peroxisome biogenesis disorder Zellweger syndrome with hepatocyte-specific elimination of the *PEX5* gene [51]. Furthermore, the *pex5Δ* mutation increased the concentrations of FFA, DAG and TAG in CR yeast (Figures 1G to I), promoting their buildup in the ER and lipid bodies (Figure 1K). CR yeast carrying the *pex5Δ* mutation also accumulated the ER-derived and lipid bodies-deposited ergosteryl esters (EE) neutral lipid species (Figure 1K).

Following a short-term exposure to exogenous FFA (palmitoleic acid or oleic acid) or DAG, wild-type (WT) cells grown under CR conditions died (Supplementary Figure 1A). The vast majority of these WT cells displayed propidium iodide (PI) positive staining characteristic of the loss of plasma membrane integrity, a hallmark event of necrotic cell death (Supplementary Figure 1B and 1C). In contrast, only a minor portion of these WT cells displayed Annexin V positive staining used to visualize the externalization of phosphatidylserine, a hallmark event of apoptotic cell death (Supplementary Figure 1B and 1C). Thus, a brief exposure of WT cells grown under CR conditions to exogenous FFA or DAG caused their necrotic, not apoptotic, death. Importantly, we found that the *pex5Δ* mutation enhances the susceptibility of CR yeast to necrotic death caused by a short-term exposure to exogenous FFA or DAG (Supplementary Figure 1A and 1C), perhaps due to the increased concentrations of endogenous FFA and DAG seen in *pex5Δ* cells under CR (Figures 1G and H).

In addition to its effect on lipid metabolism and lipid-induced necrotic cell death, the *pex5Δ* mutation also altered mitochondrial morphology and oxidation-reduction processes in mitochondria of CR yeast. In fact, this mutation caused the fragmentation of a tubular mitochondrial network into individual mitochondria under CR conditions (Figures S2A and S2B). Furthermore, in CR yeast the *pex5Δ* mutation 1) greatly reduced the rate of oxygen consumption by mitochondria (Supplementary Figure 2C); 2) substantially decreased the mito-chondrial membrane potential (Supplementary Figure 2D); and 3) diminished the level of intracellular ROS (Supplementary Figure 2E), known to be generated mostly as by-products of mitochondrial respiration [10,52]. Interestingly, all these mitochondrial abnormalities in *pex5Δ* yeast under CR were reminiscent of changes in mitochondrial morpholo-gy and functions seen in mice with hepatocyte-specific elimination of the *PEX5* gene, a model for the peroxi-some biogenesis disorder Zellweger syndrome [51].

Besides its profound effect on lipid metabolism, lipid-induced necrosis, mitochondrial morphology and functions, the *pex5Δ* mutation also 1) reduced the resistance of chronologically aging CR yeast to chronic oxidative, thermal and osmotic stresses (Supplementary Figure 3A); 2) sensitized CR yeast to death that was initiated in response to a short-term exposure to exogenous hydrogen peroxide or acetic acid (Supplementary Figure 3B) and that is known to be caused by mitochondria-controlled apoptosis [53,54]; and 3) elevated the frequencies of deletion and point mutations in mitochondrial and nuclear DNA of CR yeast (Figures S3C to S3E).

**Figure 2.**
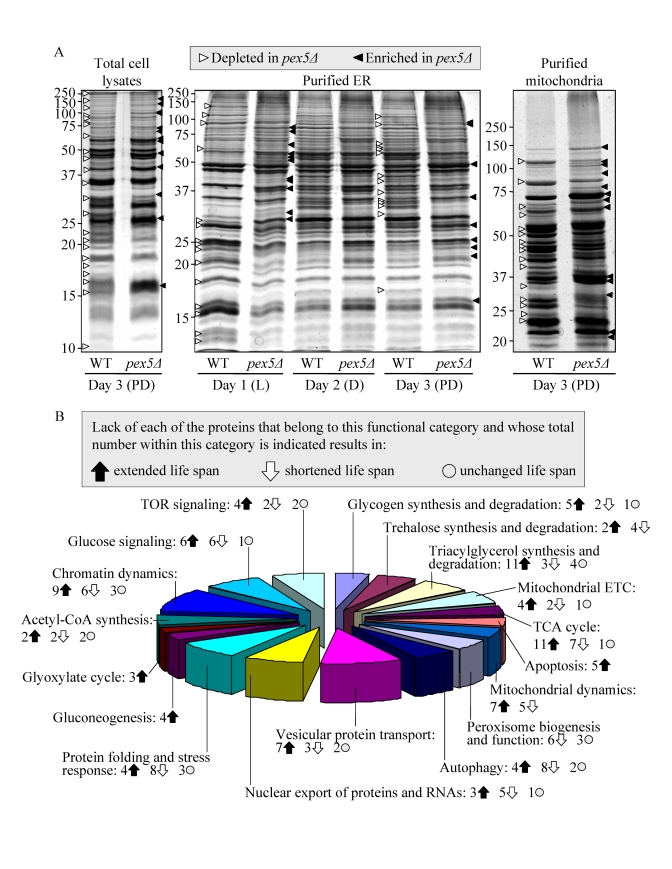
The *pex5Δ* mutation alters the abundance of many proteins recovered in total cell lysates, purified ER and mitochondria of CR yeast. (**A**) The spectra of proteins recovered in total cell lysates, purified ER and mitochondria of WT and *pex5Δ* cells that were grown under CR on 0.2% glucose and taken for analyses at the indicated time-points. (**B**) Functional categories of proteins that were enriched or depleted in the total cell lysate, ER and mitochondria of *pex5Δ* cells (as compared to WT cells) under CR conditions. Lack of 91 of these proteins increased the CLS of yeast under CR, suggesting their essential pro-aging role in longevity regulation when calorie supply is limited.

The profound changes in cell morphology and physiology, stress resistance, susceptibility to lipid-induced necrosis and mitochondria-controlled apoptosis, and stability of nuclear and mitochondrial DNA seen in *pex5Δ* yeast under CR conditions coincided with considerable changes in their proteome. Indeed, our mass spectrometry-based quantitative proteomic analysis of proteins recovered in total cell lysates as well as in purified ER and mitochondria revealed that the *pex5Δ* mutation altered the abundance of many proteins (Figure 2A). Protein species that were depleted or enriched in the total cell lysate, ER and mitochondria of *pex5Δ* yeast grown under CR conditions included proteins involved in a number of cellular processes (Figure 2B). Importantly, lack of 91 of these proteins increased the CLS of yeast under CR (Figure 2B), suggesting their essential pro-aging role in longevity regulation when calorie supply is limited. Noteworthy, 58 of the genes encoding these proteins and termed gerontogenes (*i.e*., the genes whose mutant alleles extend life span; [55]) have not been previously known as being critical for defining the CLS of yeast. The identities of protein species that were depleted or enriched in *pex5Δ* yeast grown under CR conditions, the extent to which their levels were altered and the names of gerontogenes identified in our functional analysis will be reported elsewhere (Goldberg et al., manuscript in preparation). Importantly, for most of these proteins (with some exceptions, see Figures S4C and S4D) the fold increase or decrease in the level of a protein enriched or depleted in *pex5Δ* was found to be in good correlation with the fold increase or decrease (respectively) in the mean CLS of a mutant strain lacking it (Figures S4A and S4B).

**Figure 3.**
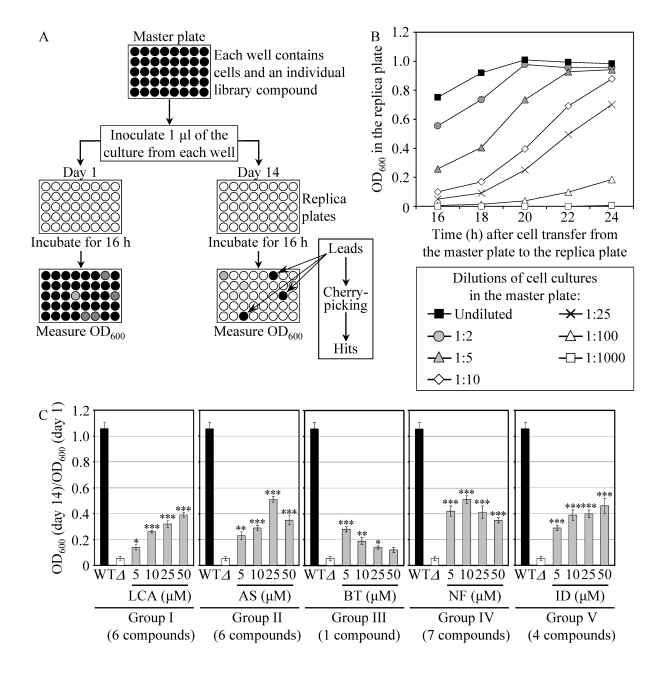
A high-throughput screen of compound libraries for small molecules that extend the CLS of yeast under CR conditions. (**A**) A microplate assay for measuring yeast CLS by monitoring optical density at 600 nm (OD_600_) was used for screening representative compounds from several commercial libraries for small molecules that extend the CLS of *pex5Δ* cells grown under CR on 0.5% glucose. (**B**) The OD_600_ of a cell culture in the replica microplate following incubation for 16 to 24 hours correlates with the number of viable cells present in this culture before it was taken from the master microplate for replica plating. (**C**) The effect of various concentrations of the identified anti-aging small molecules on the CLS of the *pex5*Δ (Δ) strain under CR conditions. The "OD_600_ at day 14/OD_600_ at day 1" ratio was used as a measure of CLS. Data are presented as means ± SEM (n = 3-5; ***p < 0.001; **p < 0.01; *p < 0.05). The anti-aging small molecules LCA, AS, BT, NF and ID belong to five chemical groups.

Altogether, these findings imply that, by impairing peroxisomal FFA oxidation and affecting lipid metabolism in the ER and lipid bodies, the *pex5Δ* mutation alters the levels of numerous pro- and anti-aging proteins and impacts many longevity-related processes, thereby shortening the CLS of yeast when calorie supply is limited. We therefore chose the short-lived *pex5Δ* strain to carry out a chemical genetic screen for anti-aging compounds that target lipid metabolism to extend CLS in yeast placed on a CR diet.

### A chemical genetic screen for small molecules that extend the CLS of yeast under CR conditions

To facilitate a high-throughput screen of compound libraries for anti-aging small molecules, we adopted a previously described microplate assay [56] for measuring CLS by monitoring optical density at 600 nm (OD_600_) (Figure 3A). In our assay, a small aliquot of the *pex5Δ* culture grown in a nutrient-rich medium containing 0.5% glucose and recovered from mid-logarithmic phase was transferred into each well of a 96-well master microplate containing the same growth medium and a compound from a commercially available library. At days 1, 7, 10 and 14 of the incubation of master microplates, a small aliquot of each culture was transferred into individual wells of a new (replica) microplate containing growth medium only. Following incubation of replica microplates for 16 hours, the OD_600_ of the culture in each well of the replica microplate was measured. Importantly, we found that under such conditions the OD_600_ of a cell culture in a well of the replica microplate correlates with the number of viable cells in the corresponding well of the master microplate (Figure 3B). To calculate survival at each time point, the OD_600_ at a particular time point was divided by the OD_600_ at day 1. By translating our microplate assay into high-throughput format and screening representative compounds from the NIH Clinical Collection, Prestwick Chemical Inc. and Sigma-LOPAC commercial libraries, we identified "lead" compounds. The subsequent "cherry-picking" analysis of these small molecules revealed "hit" compounds that in our microplate assay reproducibly extended the CLS of *pex5Δ*. Using the web-based eMolecules searching engine, we identified commercially available structural analogs of the hit compounds and then tested their life-extending efficacy in our microplate assay for measuring the CLS of *pex5Δ*. By screening the total of approximately 19,000 representative compounds from seven commercial libraries, we identified 24 small molecules that greatly extend the CLS of *pex5Δ* under CR and belong to 5 chemical groups (Figure 3C). Group I consisted of 6 bile acids, including lithocholic acid (LCA), deoxycholic acid (DCA), chenodeoxycholic acid (CDCA), cholic acid (CA), dehydrocholic acid (DHCA) and hyodeoxycholic acid (HDCA) (Figures 3C and S5). Noteworthy, the anti-aging efficacy of these bile acids correlated with their hydrophobicity. In fact, LCA - the most hydrophobic bile acid species [57] - displayed the highest ability to delay chronological aging of *pex5Δ* under CR conditions in the microplate assay (Supplementary Figure 5). The identities of small molecules that belong to groups II to V (Figure 3C) of the anti-aging compounds identified in our screen and the structure-activity analysis of their life-extending potential will be reported elsewhere (Goldberg et al., manuscript in preparation).

Noteworthy, none of the small molecules that has been shown to extend CLS (*i.e*., caffeine, methionine sulfoximine, rapamycin and spermidine; Supplementary Table 1; [56,58,59]) and/or RLS (*i.e*., rapamycin and resveratrol; Supplementary Table 1; [27,31]) in yeast has been identified in our screen for compounds capable of increasing the CLS of *pex5Δ* under CR. Furthermore, none of these currently known life-extending molecules is structurally related to the anti-aging compounds that we revealed. Thus, it is likely that LCA and all other novel anti-aging compounds identified in our screen target longevity-related cellular processes that are not modulated by the presently known anti-aging small molecules. Because our screen was aimed at identifying compounds that extend yeast longevity by targeting lipid metabolism, it is conceivable that the age-related dynamics of TAG, FFA and DAG is one of such cellular processes.

### Pharmacophore modeling of the anti-aging potential of bile acids

**Figure 4.**
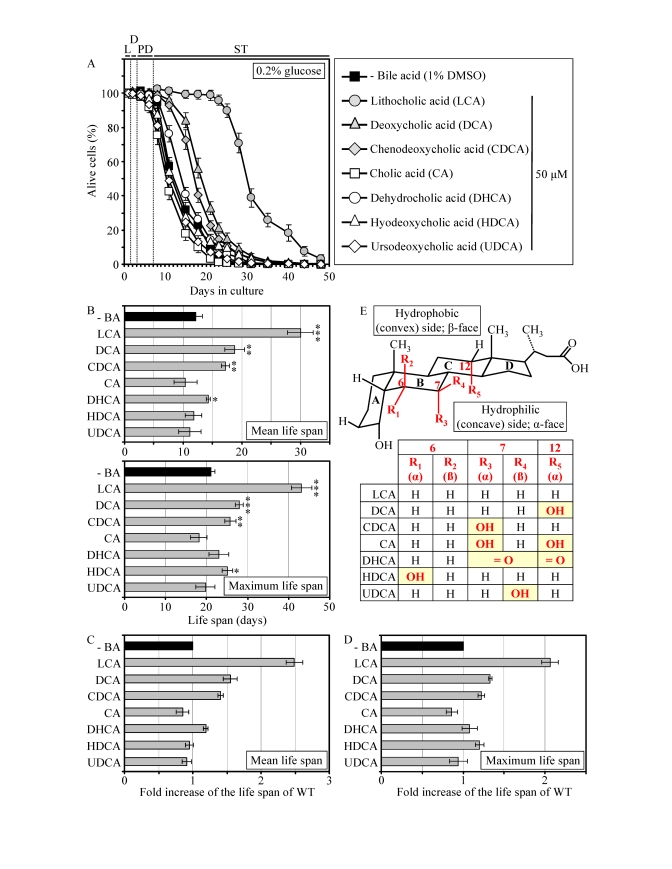
LCA and some other bile acids extend the CLS of WT strain under CR conditions. (**A** - **D**) Effect of various bile acids on survival (**A**) and on the mean and maximum life spans (**B** - **D**) of chronologically aging WT strain grown under CR conditions on 0.2% glucose. Data are presented as means ± SEM (n = 3-28; ***p < 0.001; **p < 0.01; *p < 0.05). (**E**) Structure and hydrophilic/hydrophobic properties of bile acids. The R1 (α), R3 (α) and R5 (α) hydroxyl groups at the positions 6, 7 and 12 in the six-member rings **B** and **C** of the steroid nucleus increase polarity of the hydrophilic (concave) side [α-face] of the nucleus by being located below the nucleus and axially to its plane. The R4 (β) hydroxyl group at the position 7 in the six-member ring B of the steroid nucleus confers polarity of the hydrophobic (convex) side [β-face] of the nucleus by being located above the nucleus and equatorially to its plane.

Similar to their effect on *pex5Δ*, some of the group I anti-aging compounds extended the CLS of WT strain under CR conditions. Specifically, LCA and two other bile acids - DCA and CDCA - increased both the mean and maximum CLS of WT yeast grown under CR on 0.2% glucose (Figures 4A to 4D). Moreover, DHCA increased only the mean CLS of WT yeast under CR at 0.2% glucose, whereas HDCA increased only their maximum CLS (Figures 4A to 4D). Akin to its highest life-extending efficacy in *pex5Δ* under CR, the most hydrophobic bile acid - LCA [57] - provided WT cells with the greatest longevity benefit when calorie supply was limited. In fact, LCA increased the mean CLS of WT strain under CR at 0.2% glucose by almost 250% and its maximum CLS by more than 200% (Figures 4A to D). Our comparative analysis of the structural differences between various bile acids and their relative life-extending efficacies revealed that the positions 6, 7 and 12 in the six-member rings B and C of the steroid nucleus are important for the anti-aging potential of a bile acid. Indeed, the ability of LCA to extend both the mean and maximum CLS of WT yeast under CR can be: 1) eliminated (with respect to the mean CLS) or greatly reduced (with respect to the maximum CLS) by attaching an α-oriented hydroxyl group at the position 6 (as in HDCA); 2) greatly reduced (with respect to both the mean and maximum CLS) by attaching an α-oriented hydroxyl group at the position 7 (as in CDCA); and 3) greatly reduced (with respect to both the mean and maximum CLS) by attaching an α-oriented hydroxyl group at the position 12 (as in DCA) (Figures 4B to E). All these modifications to the structure of LCA increase polarity of the hydrophilic (concave) side [α-face] of the steroid nucleus by positioning a hydroxyl group below the nucleus and axially to its plane (Figure 4E). Furthermore, the anti-aging potential of LCA can be abolished by attaching a β-oriented hydroxyl group at the position 7 (as in UDCA), thereby conferring polarity to the hydrophobic (convex) side [β-face] of the steroid nucleus by positioning a hydroxyl group above the nucleus and equatorially to its plane (Figures 4B to E).

Moreover, the simultaneous attachments of two α-oriented hydroxyl groups (as in CA) or two keto groups (as in DHCA) at the positions 7 and 12 eliminated the ability of LCA to extend both the mean and maximum CLS of WT yeast under CR (Figures 4B to E). Altogether, the results of our pharmacophore modeling of the anti-aging potential of bile acids imply that the maintenance of the minimal polarity of both the hydrophilic (concave) and hydrophobic (convex) sides of the steroid nucleus - by avoiding the presence of polar substituents at the positions 6, 7 and 12 - is mandatory for the extreme life-extending efficacy of LCA under CR conditions. Such stringent structural requirements are consistent with a target specificity of LCA action as an anti-aging small molecule.

### LCA extends the CLS of WT yeast under both CR and non-CR conditions, although to a different extent

**Figure 5.**
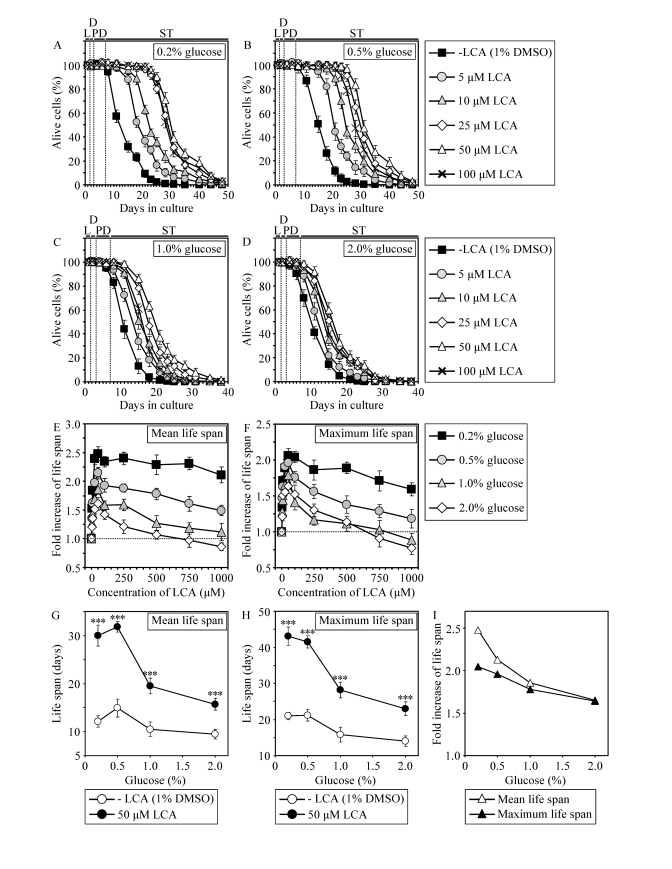
In chronologically aging WT yeast, the life-extending efficacy of LCA under CR exceeds that under non-CR conditions. (**A** - **F**) Effect of various concentrations of LCA on survival (**A** - **D**) and on the fold increase in the mean (**E**) or maximum (**F**) life span of chronologically aging WT strain cultured in medium initially containing 0.2%, 0.5%, 1% or 2% glucose. Data are presented as means ± SEM (n = 3-28). (**G** - **I**) Effect of 50 μM LCA on the mean or maximum CLS of WT yeast cultured in medium initially containing 0.2%, 0.5%, 1% or 2% glucose. Data are presented as means ± SEM (n = 12-28; ***p < 0.001).

If added to growth medium at the time of cell inoculation, LCA increased both the mean and maximum CLS of WT strain not only under CR at 0.2% or 0.5% glucose (Figures 5A, 5B and 5G - 5I) but also under non-CR conditions administered by culturing yeast in medium initially containing 1% or 2% glucose (Figures 5C, 5D and 5G - 5I). At any tested concentration of glucose in growth medium, LCA displayed the greatest beneficial effect on both the mean and maximum CLS of WT strain if used at a final concentration of 50 μM (Figures 5E and 5F). It should be stressed that the life-extending efficacy of 50 μM LCA under CR exceeded that under non-CR conditions, being inversely proportional to the concentration of glucose in growth medium and thus in correlation with the extent of calorie supply limitation (Figures 5G to 5I). Importantly, although 50 μM LCA displayed a profound effect on CLS, it did not cause significant changes in growth of WT strain at any tested concentration of glucose in medium. In fact, both growth rate in logarithmic phase and time prior to entry into stationary (ST) phase were similar for WT cells cultured in medium with or without LCA (Supplementary Figure 6).

### LCA extends the CLS of WT yeast under CR by modulating a compendium of longevity-related processes

Our chemical genetic screen identified LCA as a compound that under CR conditions extends the CLS of *pex5Δ*, a prematurely aging mutant strain displaying profound changes in lipid metabolism, lipid-induced necrotic cell death, mitochondrial morphology and functions, stress resistance, mitochondria-controlled apoptosis, and stability of nuclear and mitochondrial DNA. We found that LCA also greatly increases the mean and maximum CLS of WT yeast limited in calorie supply. This finding prompted us to investigate how the exposure of WT cells to LCA under CR conditions influences a compendium of longevity-related processes impaired in *pex5Δ*.

**Figure 6.**
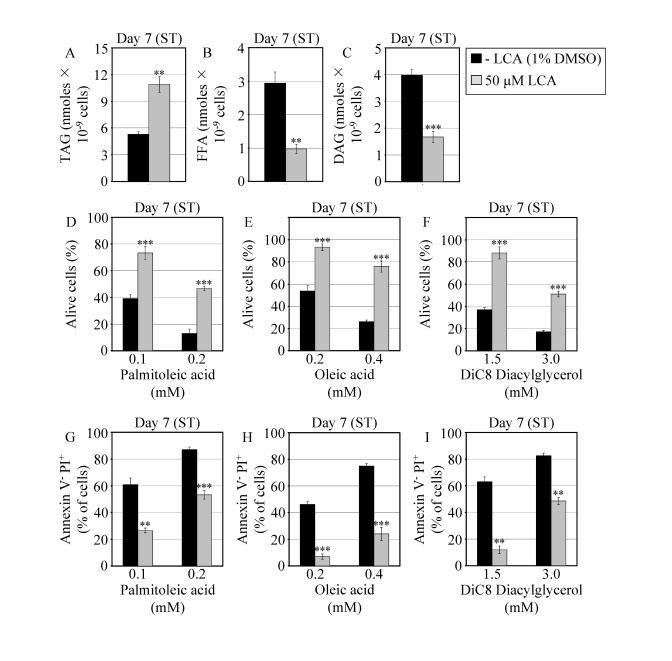
In chronologically aging WT yeast that entered the non-proliferative stationary (ST) phase under CR, LCA alters the levels of lipids and protects cells from lipid-induced necrotic death. (**A **- **C**) Levels of triacylglycerols (TAG) and free fatty acids (FFA) measured by quantitative mass spectrometry (**A** and **B**, respectively) and of diacylglycerols (DAG) monitored by TLC (**C**) in WT cells grown in medium with or without LCA. (**D** - **F**) Viability of WT cells pre-grown in medium with or without LCA and then treated for 2 h with palmitoleic acid (**D**), oleic acid (**E**) or DiC8 diacylglycerol (**F**). (**G** - **I**) Percent of WT cells (pre-grown in medium with or without LCA) that following their treatment with palmitoleic acid (G), oleic acid (H) or DiC8 diacylglycerol (**I**) displayed Annexin V negative and PI positive (Annexin V^-^ and PI^+^) staining characteristic of necrotic cell death. Data are presented as means ± SEM (n = 3-9; ***p < 0.001; **p < 0.01). WT cells grown on 0.2% glucose in the presence or absence of LCA were taken for analyses at day 7, when they reached reproductive maturation by entering into ST phase.

Consistent with its sought-after effect on lipid metabolism in the ER, lipid bodies and peroxisomes, LCA elevated the concentration of TAG in WT cells that entered the non-proliferative ST phase under CR at 0.2% glucose (Figure 6A). Furthermore, under these conditions LCA also substantially reduced the intracellular levels of FFA and DAG in WT yeast that reached reproductive maturation by entering into ST phase (Figures 6B and 6C). Moreover, LCA greatly reduced the susceptibility of reproductively mature WT cells under CR to necrotic cell death that was caused by a short-term exposure to exogenous FFA or DAG and defined by Annexin V^-^/PI^+^ staining (Figures 6D to 6I).

**Figure 7.**
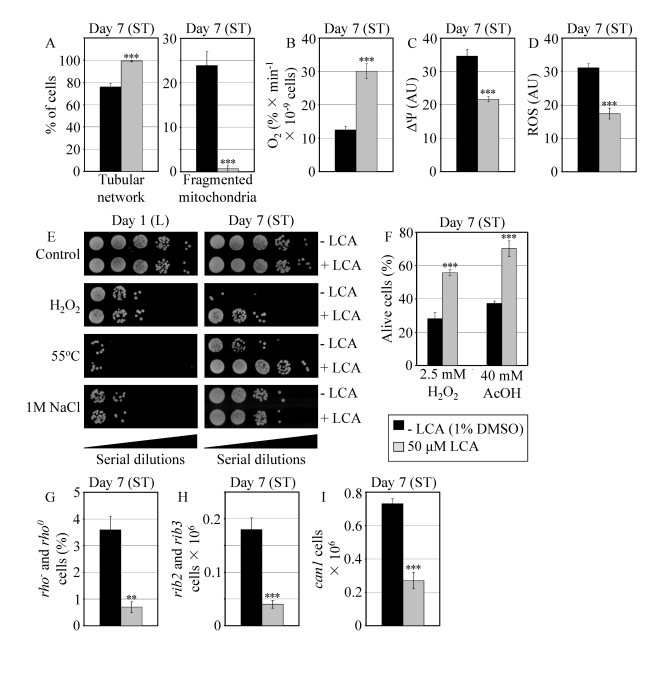
In reproductively mature WT yeast that entered the non-proliferative stationary (ST) phase under CR, LCA modulates mitochondrial morphology and functions, enhances stress resistance, attenuates mitochondria-controlled apoptosis, and increases stability of nuclear and mitochondrial DNA. (**A**) Percent of WT cells grown in medium with or without LCA and exhibiting a tubular mitochondrial network or fragmented mitochondria. Mitochondria were visualized by indirect immunofluorescence microscopy using monoclonal anti-porin primary antibodies and Alexa Fluor 568-conjugated goat anti-mouse IgG secondary antibodies. (**B** - **D**) Oxygen consumption by WT cells grown in medium with or without LCA (**B**), their mitochondrial membrane potential ΔΨ (**C**) and their ROS levels (D). ΔΨ and ROS were visualized in living cells by fluorescence microscopy using fluorescent dyes Rhodamine 123 or Dihydrorhodamine 123, respectively. (**E**) The resistance of WT cells pre-grown in medium with or without LCA to chronic oxidative, thermal and osmotic stresses. (**F**) Viability of WT cells pre-grown in medium with or without LCA and then treated for 1 h with hydrogen peroxide or acetic acid (AcOH) to induce mitochondria-controlled apoptosis. (**G- I**) The frequencies of *rho^-^* and *rho^0^*mutations in mitochondrial DNA (**G**), *rib2* and *rib3* mutations in mitochondrial DNA (**H**), and of *can1* (Can^r^) mutations in nuclear DNA (I) of WT cells grown in medium with or without LCA. Data in **A** - **D** and **F** - **I** are presented as means ± SEM (n = 4-17; ***p < 0.001; **p < 0.01). WT cells grown on 0.2% glucose in the presence or absence of LCA were taken for analyses at day 7, when they reached reproductive maturation by entering into ST phase.

The exposure of reproductively mature WT cells to LCA under CR conditions also influenced other longevity-related processes impaired in *pex5Δ*, including those confined to mitochondria. Indeed, in WT cells that entered the non-proliferative ST phase under CR at 0.2% glucose, LCA 1) attenuated the fragmentation of a tubular mitochondrial network into individual mitochondria (Figure 7A); 2) elevated the rate of oxygen consumption by mitochondria (Figure 7B); 3) reduced the mitochondrial membrane potential (Figure 7C); and 4) decreased the level of intracellular ROS (Figure 7D) known to be generated mainly in mitochondria [10,52].

Moreover, in WT yeast that under CR conditions reached reproductive maturation by entering into ST phase, LCA 1) enhanced cell resistance to oxidative and thermal (but not to osmotic) stresses (Figure 7E); 2) reduced cell susceptibility to death triggered by a short-term exposure to exogenous hydrogen peroxide or acetic acid (Figure 7F) known to be caused by mitochondria-controlled apoptosis [53,54]; and 3) decreased the frequencies of deletions and point mutations in mitochondrial and nuclear DNA (Figures 7G to I).

### LCA extends yeast CLS independent of TOR, by modulating housekeeping longevity assurance pathways

Our chemical genetic screen was aimed at identifying small molecules that can increase the CLS of yeast under CR by modulating housekeeping longevity pathways. Such pathways may regulate yeast longevity irrespective of the number of available calories and may not necessarily overlap (or may only partially overlap) with the adaptable longevity pathways that are under the stringent control of calorie availability. In chronologically aging yeast, the TOR and cAMP/PKA signaling pathways are the two adaptable longevity pathways that govern the life-extending effect of CR (Figure 10A) [5,6,60-62].

Reduction of the Tor1p protein kinase activity in yeast placed on a CR diet or exposed to rapamycin prevents inhibitory phosphorylation of Atg13p, a key positive regulator of autophagy, thereby activating this essential anti-aging process (Figure 10A) [63,64]. Under CR conditions or in response to rapamycin, Tor1p is also unable to phosphorylate and activate the nutrient-sensory protein kinase Sch9p [60,65]. The resulting inhibition of the Sch9p kinase activity suppresses its ability to attenuate protein synthesis in mitochondria, thus turning on this essential anti-aging process [61].

Furthermore, by inhibiting the Sch9p kinase activity, CR restrains Sch9p from activating protein synthesis in the cytosol, thereby slowing down this essential pro-aging process [60,62,65]. Moreover, the attenuation of the Sch9p kinase activity in CR yeast prevents the retention of Rim15p in the cytosol, hence allowing this nutrient-sensory protein kinase to enter the nucleus where it orchestrates an anti-aging transcriptional program by activating the stress response transcriptional activators Msn2p, Msn4p and Gis1p [58,62]. The longevity benefit associated with CR in chronologically aging yeast is also due to the attenuation of signaling through the cAMP/PKA pathway, which is driven by glucose deprivation [5,6,62]. By preventing inhibitory phosphorylation of Atg13p, the reduction of the PKA kinase activity in CR yeast results in activation of autophagy (Figure 10A) [63,66]. In addition, by inhibiting the PKA kinase activity, CR suppresses the ability of PKA to activate protein synthesis in the cytosol [62]. Moreover, reduced PKA kinase activity in CR yeast enables nuclear import of Msn2p and Msn4p, thus turning on an anti-aging transcriptional program driven - in a Rim15p-dependent fashion - by these two transcriptional activators [27,62,67]. Noteworthy, the kinase activity of the cytosolic pool of Rim15p is inactivated through PKA-dependent phosphorylation (Figure 10A) [62]. Although some of the Rim15p phosphorylation targets are involved in longevity regulation and reside outside the nucleus [68], a role of such phosphorylation in the life-extending effect of CR in yeast remains to be established.

**Figure 8.**
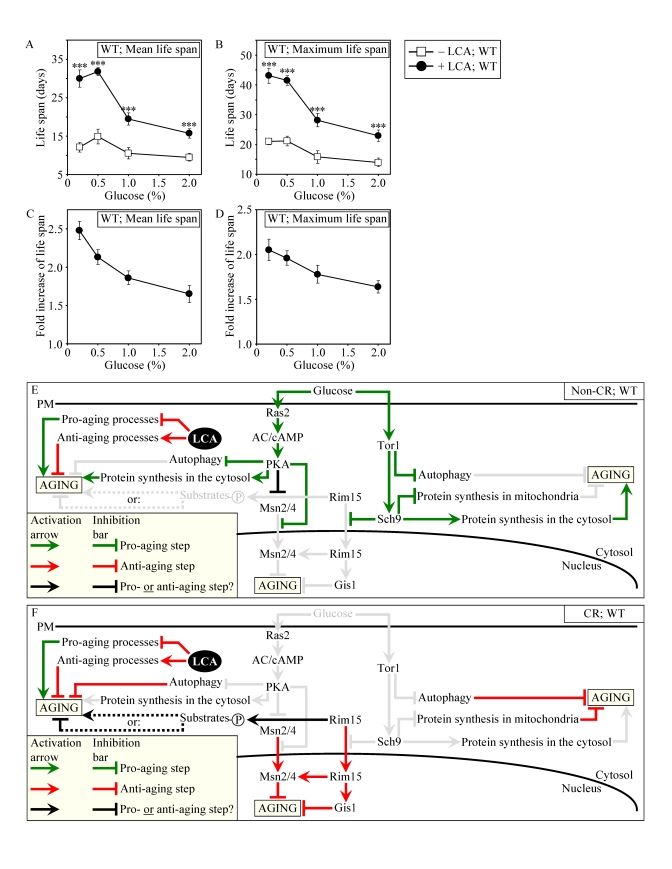
LCA increases the CLS of WT strain to the highest extent under CR conditions. (**A** and **B**) Effect of LCA on the mean (**A**) and maximum (**B**) life spans of chronologically aging WT strain. Data are presented as means ± SEM (n = 12-28; ***p < 0.001). (**C** - **E**) Effect of LCA on the fold increase in the mean (**C**) or maximum (**D**) life span of chronologically aging WT strain. Data are presented as means ± SEM (n = 12-28). Cells in **A** to **D** were cultured in medium initially containing 0.2%, 0.5%, 1% or 2% glucose in the presence of LCA (50 μM) or in its absence. Survival data are provided in Supplementary Figure 9. (**E** and **F**) Outline of pro- and anti-aging processes that are controlled by the TOR and/or cAMP/PKA signaling pathways and are modulated by LCA in WT cells grown under non-CR (**E**) or CR (**F**) conditions. Activation arrows and inhibition bars denote pro-aging processes (displayed in green color), anti-aging processes (displayed in red color) or processes whose role in longevity regulation is presently unknown (displayed in black color). Doted lines denote hypothetical processes. Abbreviations: PM, plasma membrane.

Our evaluation of the life-extending efficacy of LCA in WT strain on a high- or low-calorie diet revealed that this compound increased CLS irrespective of the number of available calories (Figures 8A and 8B). Intriguingly, the extent to which LCA extended longevity was highest under CR conditions (Figures 8C and 8D), when the pro-aging processes modulated by the adaptable TOR and cAMP/PKA pathways are suppressed and the anti-aging processes are activated (Figure 8F). The life-extending efficacy of LCA in CR yeast significantly exceeded that in yeast on a high-calorie diet (Figures 8C and 8D), in which the adaptable TOR and cAMP/PKA pathways greatly activate the pro-aging processes and suppress the anti-aging processes (Figure 8E). Altogether, these findings suggest that, consistent with its sought-after effect on a longevity signaling network, LCA mostly targets certain housekeeping longevity assurance pathways that do not overlap (or only partially overlap) with the adaptable TOR and cAMP/PKA pathways modulated by calorie availability (Figures 8E and 8F).

**Figure 9.**
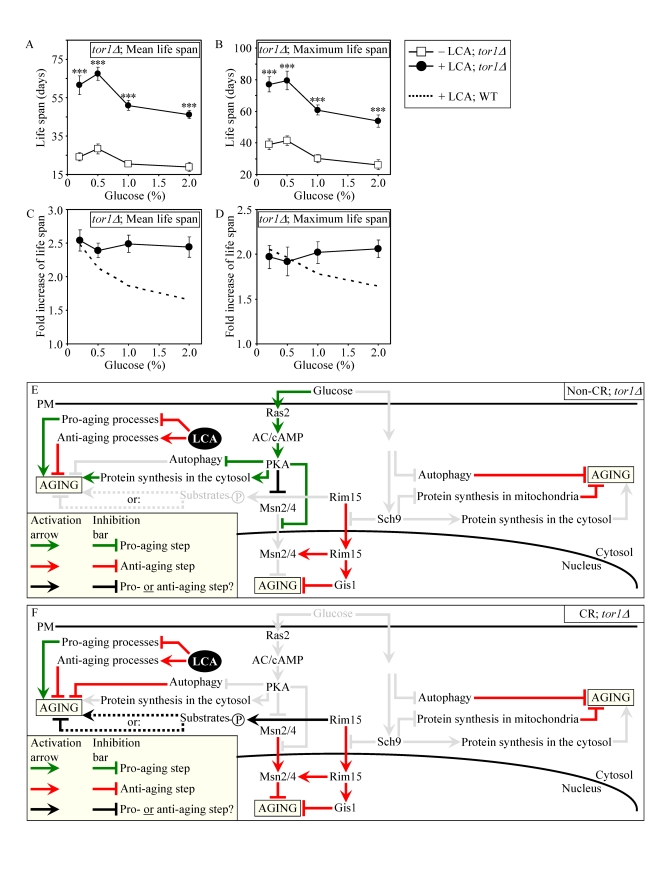
Lack of Tor1p does not impair the life-extending effect of LCA and abolishes the dependence of the anti-aging efficacy of LCA on the number of available calories. (**A** and **B**) Effect of LCA on the mean (**A**) and maximum (**B**) life spans of chronologically aging *tor1Δ* strain. Data are presented as means ± SEM (n = 4-7; ***p < 0.001). (**C** and **D**) Effect of LCA on the fold increase in the mean (**C**) or maximum (**D**) life spans of chronologically aging *tor1Δ* and WT strains. Data are presented as means ± SEM (n = 4-7). Cells in **A** to **D** were cultured in medium initially containing 0.2%, 0.5%, 1% or 2% glucose in the presence of LCA (50 μM) or in its absence. Survival data are provided in Supplementary Figure 10. (**E** and **F**) Outline of pro- and anti-aging processes that are controlled by the TOR and/or cAMP/PKA signaling pathways and are modulated by LCA in *tor1Δ*cells grown under non-CR (**E**) or CR (**F**) conditions.

Consistent with our assumption that LCA extends longevity not by modulating the adaptable TOR pathway (Figures 9E and 9F), lack of Tor1p did not impair the life-extending efficacy of LCA under CR (Figures 9A to 9D). Importantly, by eliminating a master regulator of this key adaptable pathway that shortens the CLS of yeast on a high-calorie diet, the *tor1Δ* mutation abolished the dependence of the anti-aging efficacy of LCA on the number of available calories. In fact, LCA extended longevity of the *tor1Δ* mutant strain to a very similar degree under CR and non-CR conditions (Figures 9C and 9D).

We next assessed how the adaptable cAMP/PKA pathway influences the life-extending efficacy of LCA in yeast on a high- or low-calorie diet. Although the *ras2Δ* mutation greatly decreases the PKA protein kinase activity by eliminating a GTP-binding protein that activates adenylate cyclase responsible for the synthesis of the PKA activator cAMP (Figures S7E and S7F) [62], it did not abolish the ability of LCA to extend CLS under CR and non-CR conditions (Figures S7A and S7B). However, the life-extending efficacy of LCA was decreased by the *ras2Δ* mutation, as compared to that seen in WT cells exposed to this compound (Figures S7C and S7D). In spite of such partial reduction of the anti-aging potential of LCA in *ras2Δ*, LCA still significantly increased its CLS under CR and non-CR conditions (Figures S7C and S7D).

**Figure 10.**
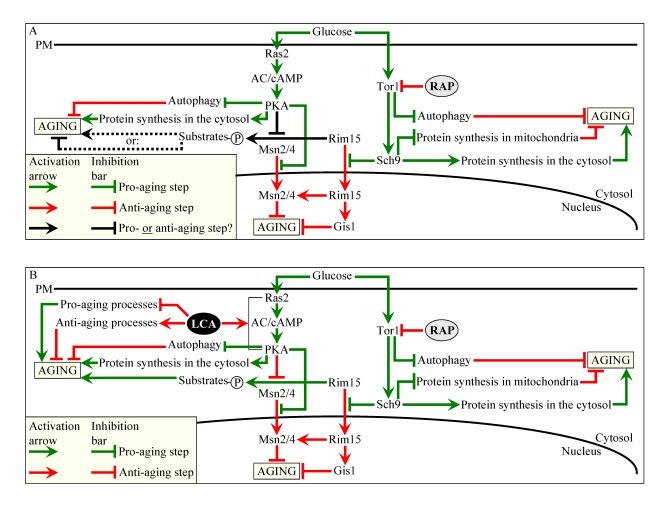
Outline of pro- and anti-aging processes that are controlled by the TOR and/or cAMP/PKA signaling pathways and are modulated by LCA or rapamycin (RAP) in chronologically aging yeast. The currently accepted (**A**) and updated, based on this study (**B**), outlines of pro- and anti-aging processes are shown. Activation arrows and inhibition bars denote pro-aging processes (displayed in green color), anti-aging processes (displayed in red color) or processes whose role in longevity regulation was unknown (displayed in black color). Doted lines denote hypothetical, until this study, processes. See text for details.

Thus, it seems that LCA extends longevity of chronologically aging yeast through two different mechanisms. Firstly, irrespective of the number of available calories, this bile acid targets certain house-keeping longevity assurance pathways that 1) inhibit some pro-aging processes and/or activate some anti-aging processes; and 2) do not overlap with the adaptable cAMP/PKA pathway modulated by calorie availability (Figure 10B). Secondly, we propose that LCA unmasks the anti-aging potential of PKA by activating PKA-dependent phosphorylation of the cytosolic pool of Rim15p (Figure 10B). Because such phosphorylation of Rim15p is known to inactivate its protein kinase activity (Figure 10A) [62], we hypothesize that, while the nuclear pool of Rim15p has a well established anti-aging function [5,6,27,62], the cytosolic pool of this nutrient-sensory protein kinase plays an essential pro-aging role by phosphorylating a compendium of proteins that promote aging only if phosphorylated (Figure 10B). Noteworthy, some of the Rim15p phosphorylation targets are involved in longevity regulation and reside outside the nucleus [68]. In our hypothesis, LCA can unmask the anti-aging potential of PKA only when PKA is activated by cAMP, *i.e.*, under non-CR conditions (Figures 8E and F). Consistent with our hypothesis on the two mechanisms underlying the anti-aging effect of LCA, lack of Ras2p only partially and to the same extent reduced the life-extending potential of LCA under both CR and non-CR conditions (Figures S7C and S7D), likely by impairing the mechanism in which LCA unmasks the anti-aging potential of PKA. The resulting inability of PKA to inhibit the proposed pro-aging role of the cytosolic pool of Rim15p in *ras2Δ* cells would make the Rim15p-dependent pro-aging mechanism constitutively active in these cells, regardless of the number of available calories or presence of LCA (Figures S7E and S7F).

The TOR and cAMP/PKA pathways converge on Rim15p whose nuclear pool plays a pivotal role in governing the life-extending effect of CR by enabling the establishment of an anti-aging transcriptional program driven by Msn2p, Msn4p and Gis1p (Figure 10A) [5,6,27,62]. Our evaluation of the life-extending efficacy of LCA in yeast lacking Rim15p further supported the notion that one of the two mechanisms underlying the anti-aging effect of this bile acid involves its ability to modulate certain housekeeping longevity assurance pathways that are not centered on Rim15p and do not overlap with the adaptable TOR and cAMP/PKA pathways. In fact, although the life-extending potential of LCA in *rim15Δ* was partially reduced (Figures S8C and S8D) due to the impairment of the Rim15p-centered mechanism of its anti-aging action (Figures S8E and S8F), LCA still significantly increased the CLS of *rim15Δ* under CR and non-CR conditions (Figures S8A to S8D). Importantly, by eliminating a key nutrient-sensory protein kinase on which the adaptable TOR and cAMP/PKA pathways converge to regulate longevity in a calorie availability-dependent fashion, the *rim15Δ* mutation abolishedthe dependence of the anti-aging efficacy of LCA on the number of available calories (Figures S8C and S8D).

## Discussion

In this study, we designed a chemical genetic screen for small molecules that increase the CLS of yeast under CR conditions by targeting lipid metabolism and modulating housekeeping longevity pathways that regulate longevity irrespective of the number of available calories. Our screen identifies LCA as one of such molecules. Our analysis of how LCA influences various longevity-related processes and how it affects the CLS of yeast mutants impaired in the adaptable TOR and cAMP/PKA longevity pathways provided important new insights into mechanisms of longevity regulation, as outlined below.

### LCA extends yeast CLS by modulating housekeeping longevity assurance processes that are not regulated by the adaptable TOR and cAMP/PKA

### signaling pathways

Our findings imply that LCA extends longevity of chronologically aging yeast by targeting two different mechanisms. One mechanism extends longevity regardless of the number of available calories. This mechanism involves the LCA-governed modulation of certain housekeeping longevity assurance pathways that do not overlap with the adaptable TOR and cAMP/PKA pathways (Figure 10B). We identify a compendium of processes that compose LCA-targeted housekeeping longevity assurance pathways. Our data provide evidence that LCA modulates these pathways by 1) suppressing the pro-aging process [39,40,50] of lipid-induced necrotic cell death, perhaps due to its observed ability to reduce the intracellular levels of FFA and DAG that trigger such death; 2) attenuating the pro-aging process [69,70] of mitochondrial fragmentation, a hallmark event of age-related cell death; 3) altering oxidation-reduction processes in mitochondria - such as oxygen consumption, the maintenance of membrane potential and ROS production - known to be essential for longevity regulation [8,10,11,71]; 4) enhancing cell resistance to oxidative and thermal stresses, thereby activating the anti-aging process [11,39,40,72,73] of stress response; 5) suppressing the pro-aging process [69,70] of mitochondria-controlled apoptosis; and 6) enhancing stability of nuclear and mitochondrial DNA, thus activating the anti-aging process [74,75] of genome maintenance. The observed pleiotropic effect of LCA on a compendium of housekeeping longevity assurance processes implies that this bile acid is a multi-target life-extending compound that increases CLS in yeast by modulating a network of the highly integrated processes that are not controlled by the adaptable TOR and cAMP/PKA pathways. The major challenge now is to define the molecular mechanisms by which LCA modulates each of these pro- and anti-aging housekeeping processes and integrates them in chronologically aging yeast.

The other mechanism underlying the life-extending effect of LCA in chronologically aging yeast increases life span only under non-CR conditions. This mechanism consists in LCA-driven unmasking of the previously unknown anti-aging potential of PKA, a key player in the adaptable cAMP/PKA pathway. We propose that LCA unveils the anti-aging potential of PKA by activating PKA-dependent phosphorylation of the cytosolic pool of Rim15p, a key nutrient-sensory protein kinase on which the adaptable TOR and cAMP/PKA pathways converge to regulate longevity in a calorie availability-dependent fashion (Figure 10B). Of note, the nuclear pool of Rim15p is well known for its anti-aging role in governing the life-extending effect of CR by enabling a pro-longevity transcriptional program driven by Msn2p, Msn4p and Gis1p (Figure 10B) [6,62]. In our hypothesis 1) unlike its nuclear pool, the cytosolic pool of Rim15p has an essential pro-aging function in phosphorylating a compendium of its cytosolic target proteins [68] some of which promote aging only if phosphorylated (Figure 10B); 2) under non-CR conditions LCA activates the PKA-dependent phosphorylation of Rim15p (Figure 10B); and 3) because the phosphorylation of Rim15p inactivates its protein kinase activity [62], the dephosphorylation of pro-aging target proteins of Rim15p in the cytosol by phosphatases inhibits the ability of these target proteins to promote aging (Figure 10B). To test the validity of our hypothesis, we are currently evaluating how genetic manipulations that alter the abundance of various extra-nuclear target proteins of Rim15p or affect their phosphorylation status influence the life-extending efficacy of LCA.

### Bile acids are beneficial to health and longevity across phyla

It should be stressed that, although we found that LCA greatly extends yeast longevity, yeast do not synthesize this or any other bile acid found in mammals [57,76]; our mass spectrometry-based analysis of the total yeast lipidome has confirmed lack of endogenous bile acids. One could envision that during evolution yeast have lost the ability to synthesize bile acids but have maintained the life-extending response to these biologically active molecules by retaining certain longevity-related processes that are sensitive to regulation by bile acids. Alternatively, one could think that during evolution yeast have developed the ability to sense bile acids produced by mammals (and/or bile acid-like lipids synthesized by worms), recognize these mildly toxic molecules as environmental stressors providing hormetic benefits and/or as indicators of the state of the environment or food supply, and then to respond by undergoing certain life-extending changes to their physiology that ultimately increase their chances of survival. It is conceivable therefore that the life-extending potential of LCA and other bile acids as well as, probably, the mechanisms underlying their anti-aging action are evolutionarily conserved.

 In fact, following their synthesis from cholesterol in the intestine, hypodermis, spermatheca and sensory neurons of worms, bile acid-like dafachronic acids (including 3-keto-LCA) are delivered to other tissues where they activate the DAF-12/DAF-16 signaling cascade that in turn orchestrates an anti-aging transcriptional program, thereby increasing the life span of the entire organism [41]. Bile acids also provide health benefits to mammals. Synthesized from cholesterol in hepatocytes of the liver, these amphipathic molecules have been for a long time considered to function only as trophic factors for the enteric epithelium and as detergents for the emulsification and absorption of dietary lipids and fat-soluble vitamins [57,76,77]. Recent years have been marked by a significant progress in our understanding of the essential role that bile acids play as signaling molecules regulating lipid, glucose and energy homeostasis and activating detoxification of xenobiotics [57,77,78]. By stimulating the G-protein-coupled receptor TGR5, bile acids activate the cAMP/PKA signaling pathway that 1) enhances energy expenditure in brown adipose tissue and muscle by stimulating mitochondrial oxidative phosphorylation and un-coupling; 2) improves liver and pancreatic function by activating the endothelial nitric oxide synthase; and 3) enhances glucose tolerance in obese mice by inducing intestinal glucagon-like peptide-1 release [57,76,78]. Furthermore, by activating the farnesoid X receptor (FXR) and several other nuclear hormone receptors inside mammalian cells, bile acids 1) modulate the intracellular homeostasis of cholesterol, neutral lipids and fatty acids; 2) regulate glucose metabolism by enhancing glycogenesis and attenuating gluconeo-genesis; and 3) stimulate clearance of xenobiotic and endobiotic toxins by activating transcription of numerous xenobiotic detoxification genes [57,76-78]. All these health-improving, beneficial metabolic effects of bile acids prevent the development of obesity following administration of high-fat diet [57,76,77]. Thus, bile acids have a great potential as pharmaceutical agents for the treatment of diabetes, obesity and various associated metabolic disorders, all of which are age-related [57,76]. Moreover, bile acids have been shown to inhibit neuronal apoptosis in experimental rodent models of neurodegenerative disorders by promoting mitochondrial membrane stability, preventing the release of cytochrome c from mitochondria,  reducing activities of various caspases, and activating the NF-κB, PI3K and MAPK survival pathways [79,80].

It should be stressed that many of the metabolic, stress response and apoptotic processes modulated by bile acids in mammals are essential for healthy aging and longevity regulation. Importantly, we found that, by modulating several of these health- and longevity-related processes in chronologically aging yeast, LCA increases their life span. Moreover, the long-lived Ghrhr^lit/lit^ mice displayed elevated levels of several bile acids and exhibited increased FXR-dependent transcription of numerous xenobiotic detoxification genes; if administered to food consumed by wild-type mice, cholic acid - one of these bile acids - mimicked the FXR-governed gene expression pattern observed in Ghrhr^lit/lit^ mice [81,82]. It has been therefore proposed that, by promoting chemical hormesis in mammals, these mildly toxic molecules with detergent-like properties may extend their longevity by acting as endobiotic regulators of aging [73,82,83].

Altogether, these findings support the notion that bile acids act as endobiotic and xenobiotic regulators of aging that are beneficial to health and longevity across phyla. A comparative analysis of the mechanisms underlying such health-improving and life-extending action of bile acids implies that these mechanisms are likely to be evolutionarily conserved.

## Methods

Yeast strains and growth conditions. The WT strain BY4742 (*MATα his3Δ1 leu2Δ0 lys2Δ0 ura3Δ0*) and single-gene-deletion mutant strains in the BY4742 genetic background (all from Open Biosystems) were grown in YP medium (1% yeast extract, 2% peptone) containing 0.2% 0.5%, 1% or 2% glucose as carbon source. Cells were cultured at 30^o^C with rotational shaking at 200 rpm in Erlenmeyer flasks at a "flask volume/medium volume" ratio of 5:1.

Chemical genetic screen for compounds that increase chronological life span (CLS). The screen was conducted at the High Throughput/Content Screening Facility at McGill University. The single-gene-deletion mutant strain *pex5Δ* (*MAT*α* his3Δ1 leu2Δ0 lys2Δ0 ura3Δ0 pex5Δ::kanMX4*) from Open Biosystems was grown in YPA0.5D medium (1% yeast extract, 2% peptone, 50 μg/ml ampicillin, 0.5% glucose). 3-μl aliquots of the *pex5Δ*culture recovered from mid-logarithmic phase at a cell titre of 2 Í 10^7^ cells/ml were aliquoted into 96-well master microplates using a Beckman Coulter high density Biomek FXII replica pinning robot. Each well of a master microplate contained 96 μl of YPA0.5D medium. 1 μl of a compound stock solution from a commercially available library (each compound at 5 mM in dimethylsulfoxide (DMSO)) was added to each well using a Beckman Coulter high density Biomek FXII replica pinning robot. Wells of a master microplate supplemented with 1% DMSO (1 μl of DMSO per a well containing 3 μl of the *pex5Δ*culture and 96 μl of YPA0.5D medium) were used as negative controls. Each master plate was created in duplicate. The master microplates were sealed and incubated without shaking at 30^o^C in a moist chamber. At days 1, 7, 10 and 14 of the incubation of master microplates, a 3-μl aliquot of each culture was transferred into individual wells of a new (replica) microplate containing 97 μl of YPA0.5D medium. Following incubation of sealed replica microplates in a moist chamber for 16 hours at 30oC (to allow for growth of cells that were still viable), the optical density at 600 nm (OD600) of the culture in each well of the replica microplate was measured using a Molecular Devices Analyst HT plate reader. To calculate survival at each time point, the OD600 at a particular time point was divided by the OD600 at day 1. "Cherry-picking" of the identified "lead" com-pounds for possible "hits" was carried out as described above, with each lead compound being used at a final concentration of 5, 10, 25 or 50 μM and assessed in triplicate for validation. Commercially available structural analogs of hit compounds were identified using the web-based eMolecules searching engine. In total, approximately 19,000 representative compounds from the BIOMOL, Chembridge, Maybridge, MicroSource Discovery, NIH Clinical Collection, Prestwick Chemical Inc. and Sigma-LOPAC commercial libraries were tested using the screen for chemical modulators of longevity.

Pharmacological manipulation of CLS. CLS analysis was performed as previously described [39]. The chenodeoxycholic (C9377), cholic (C1129), dehydrocholic (D3750), deoxycholic (D2510), hyodeoxycholic (H3878), lithocholic (L6250) and ursodeoxycholic (U5127) bile acids were from Sigma. Their stock solutions in DMSO were made on the day of adding each of these compounds to cell cultures. Compounds were added to growth medium at the indicated concentration immediately following cell inoculation. The final concentration of DMSO in yeast cultures supplemented with a bile acid (and in the corresponding control cultures supplemented with drug vehicle) was 1% (v/v).

Miscellaneous procedures. Fluorescence [39], immuno-fluorescence [39] and electron [84] microscopies followed by morphometric analyses of the resulting images have been described elsewhere. Extraction of lipids and their separation, identification and quantitation with the help of TLC were performed according to established procedures [84]. Mass spectrometric identification and quantitation of various lipid species were carried as previously described [85]. Subcellular fractionation and organelle purification,cell viability and stress resistance assays, oxygen consumption assay, the measurement of the frequencies of spontaneous point and deletion mutations in mitochondrial and nuclear DNA, total cell lysates preparation, and mass spectrometric identification and quantitation of proteins were performed according to established procedures [39].

## Supplementary data

Supplementary Figure 1 The *pex5Δ* mutation enhances the susceptibility of CR yeast to necrotic death caused by a short-term exposure to exogenous lipids. (A) Viability of WT and *pex5Δ* cells treated for 2 h with palmitoleic acid, oleic acid or DiC8 diacylglycerol. (B) Fluorescence microscopy of WT yeast treated for 2 h with 0.25 mM palmitoleic acid, 0.6 mM oleic acid or 4.0 mM DiC8 diacylglycerol. Cells were co-stained with 1) Annexin V for visualizing the externalization of phosphatidylserine, a hallmark event of apoptosis; and 2) propidium iodide (PI) for visualizing the loss of plasma membrane integrity, a hallmark event of necrosis. (C) Percent of WT and *pex5Δ* cells that following their treatment with palmitoleic acid, oleic acid or DiC8 diacylglycerol displayed Annexin V negative and PI positive (Annexin V- and PI+) staining characteristic of necrotic cell death. Data in A and C are presented as means ± SEM (n = 3-5; ***p < 0.001; **p < 0.01; *p < 0.05). Prior to their exposure to exogenous lipids, CR yeast were grown for 2 days on 0.2% glucose. Abbreviations: DIC, differential interference contrast.

Supplementary Figure 2 The *pex5Δ* mutation alters mitochondrial morphology and functions in CR yeast. (A) Morphology of mitochondria in WT and *pex5Δ *cells. Mitochondria were visualized by indirect immunofluorescence microscopy using monoclonal anti-porin primary antibodies and Alexa Fluor 568-conjugated goat anti-mouse IgG secondary antibodies. (B) Percent of WT and *pex5Δ *cells exhibiting a tubular mitochondrial network or fragmented mitochondria. (C - E) Oxygen consumption (C) by WT and *pex5Δ *cells, their mitochondrial membrane potential ΔΨ (D) and their ROS levels (E). ΔΨ and ROS were visualized in living cells by fluorescence microscopy using fluorescent dyes Rhodamine 123 or Dihydrorhodamine 123, respectively. CR yeast grown on 0.2% glucose were taken for analyses at the indicated time-points. Data in B - E are presented as means ± SEM (n = 4-15; ***p < 0.001; **p < 0.01; *p < 0.05). Abbreviation: D, diauxic growth phase.

Supplementary Figure 3 **The* pex5Δ* mutation reduces the resistance of CR yeast to stresses, sensitizes them to exogenously induced apoptosis and elevates the frequencies of mutations in their mitochondrial and nuclear DNA. **(A) The resistance of WT and *pex5Δ* to chronic oxidative, thermal and osmotic stresses. (B) Viability of WT and *pex5Δ* cells treated for 1 h with hydrogen peroxide or acetic acid (AcOH) to induce mitochondria-controlled apoptosis. (C - E) The frequencies of *rho-* and *rho0* deletion mutations in mitochondrial DNA (C), *rib2* and *rib3* point mutations in mitochondrial DNA (D), and of *can1* point mutations in nuclear DNA (E) of WT and *pex5Δ* cells. CR yeast grown on 0.2% glucose were taken for analyses at the indicated time-points. Data in B to E are presented as means ± SEM (n = 6-9; ***p < 0.001; **p < 0.01; *p < 0.05). Abbreviations: AcOH, acetic acid; D, diauxic growth phase; L, logarithmic growth phase; PD, post-diauxic growth phase.

Supplementary Figure 4 **For many proteins, the fold increase or decrease in the level of a protein enriched or depleted in *pex5Δ* yeast under CR correlates with the fold increase or decrease (respectively) in the mean CLS of a mutant strain lacking it. **Plots comparing the fold increase or decrease in the levels of proteins enriched or depleted in *pex5Δ* yeast when calorie supply is limited and the fold increase or decrease (respectively) in the mean CLS of the single-gene-deletion mutant strains that lack these proteins and grow under CR. Each point shows the data for a single protein and a mutant strain that lacks it. Data are presented only for proteins whose levels were increased or decreased by more than 50% in *pex5Δ* cells (as compared to WT cells) grown under CR on 0.2% glucose. Linear regression is shown.

Supplementary Figure 5 The life-extending potential of a bile acid correlates with its hydrophobicity. In the microplate assay, lithocholic acid - the most hydrophobic bile acid species - displays the greatest ability to extend the CLS of the short-lived *pex5*Δ mutant strain under CR conditions. The effect of various concentrations of different bile acids on the CLS of the short-lived *pex5*Δ mutant strain grown under CR on 0.5% glucose is shown. The "OD600 at day 14/OD600 at day 1" ratio was used as a measure of CLS. Data are presented as means ± SEM (n = 3-5; ***p < 0.001; **p < 0.01; *p < 0.05).

Supplementary Figure 6 LCA does not cause significant changes in growth pattern of wild-type (WT) strain at any tested concentration of glucose in medium. Kinetics of growth for WT strain in medium initially containing 0.2%, 0.5%, 1.0% or 2.0% glucose in the presence of LCA (50 μM ) or in its presence. Each plot shows a representative experiment repeated 4-7 times in triplicate with similar results. Abbreviations: D, diauxic growth phase; L, logarithmic growth phase; PD, post-diauxic growth phase; ST, stationary growth phase.

Supplementary Figure 7 In spite of a partial reduction in the life-extending efficacy of LCA in yeast lacking Ras2p, LCA still significantly increases their mean and maximum life spans under CR and non-CR conditions. (A and B) Effect of LCA on the mean (A) and maximum (B) life spans of chronologically aging *ras2Δ* strain. Data are presented as means ± SEM (n = 4-7; ***p < 0.001; **p < 0.01; *p < 0.05). (C and D) Effect of LCA on the fold increase in the mean (C) or maximum (D) life spans of chronologically aging *ras2Δ* and WT strains. Data are presented as means ± SEM (n = 4-7). Cells in A to D were cultured in medium initially containing 0.2%, 0.5%, 1% or 2% glucose in the presence of LCA (50 μM) or in its absence. Chronological survival data for *ras2Δ* strain are provided in Supplementary Figure 11. (E and F) Outline of pro- and anti-aging processes that are controlled by the TOR and/or cAMP/PKA signaling pathways and are modulated by LCA in *ras2Δ* cells grown under non-CR (E) or CR (F) conditions. Activation arrows and inhibition bars denote pro-aging processes (displayed in green color) or anti-aging processes (displayed in red color). Abbreviations: PM, plasma membrane.

Supplementary Figure 8 Although the life-extending efficacy of LCA in yeast lacking Rim15p is partially reduced, LCA still significantly increases their mean and maximum life spans under CR and non-CR conditions. (A and B) Effect of LCA on the mean (A) and maximum (B) life spans of chronologically aging *rim15Δ* strain. Data are presented as means ± SEM (n = 5-7; ***p < 0.001; **p < 0.01). (C and D) Effect of LCA on the fold increase in the mean (C) or maximum (D) life spans of chronologically aging *rim15Δ* and WT strains. Data are presented as means ± SEM (n = 5-7). Cells in A to D were cultured in medium initially containing 0.2%, 0.5%, 1% or 2% glucose in the presence of LCA (50 μM) or in its absence. Chronological survival data for *rim15Δ* strain are provided in Supplementary Figure 12. (E and F) Outline of pro- and anti-aging processes that are controlled by the TOR and/or cAMP/PKA signaling pathways and are modulated by LCA in *rim15Δ* cells grown under non-CR (E) or CR (F) conditions. Activation arrows and inhibition bars denote pro-aging processes (displayed in green color) or anti-aging processes (displayed in red color). Abbreviations: PM, plasma membrane.

Supplementary Figure 9 Chronological survival data for WT strain cultured in medium initially containing 0.2%, 0.5%, 1% or 2% glucose in the presence of LCA (50 μM) or in its absence. Dataset for Figure 8.

Supplementary Figure 10 Chronological survival data for *tor1Δ* strain cultured in medium initially containing 0.2%, 0.5%, 1% or 2% glucose in the presence of LCA (50 μM) or in its absence. Dataset for Figure 9.

Supplementary Figure 11 Chronological survival data for *ras2Δ* strain cultured in medium initially containing 0.2%, 0.5%, 1% or 2% glucose in the presence of LCA (50 μM) or in its absence. Dataset for Supplementary Figure 7.

Supplementary Figure 12 Chronological survival data for *rim15Δ* strain cultured in medium initially containing 0.2%, 0.5%, 1% or 2% glucose in the presence of LCA (50 μM) or in its absence. Dataset for Supplementary Figure 8.

Supplementary Table 1
