# Supplementary figures and images for: Chemical genetic screen identifies lithocholic acid as an anti-aging compound that extends yeast chronological life span in a TOR-independent manner, by modulating housekeeping longevity assurance processes
Source: Aging (Albany NY). 2010 Jul 7;2(7):393–414. doi: 10.18632/aging.100168 (PMC2933888; doi:10.18632/aging.100168)

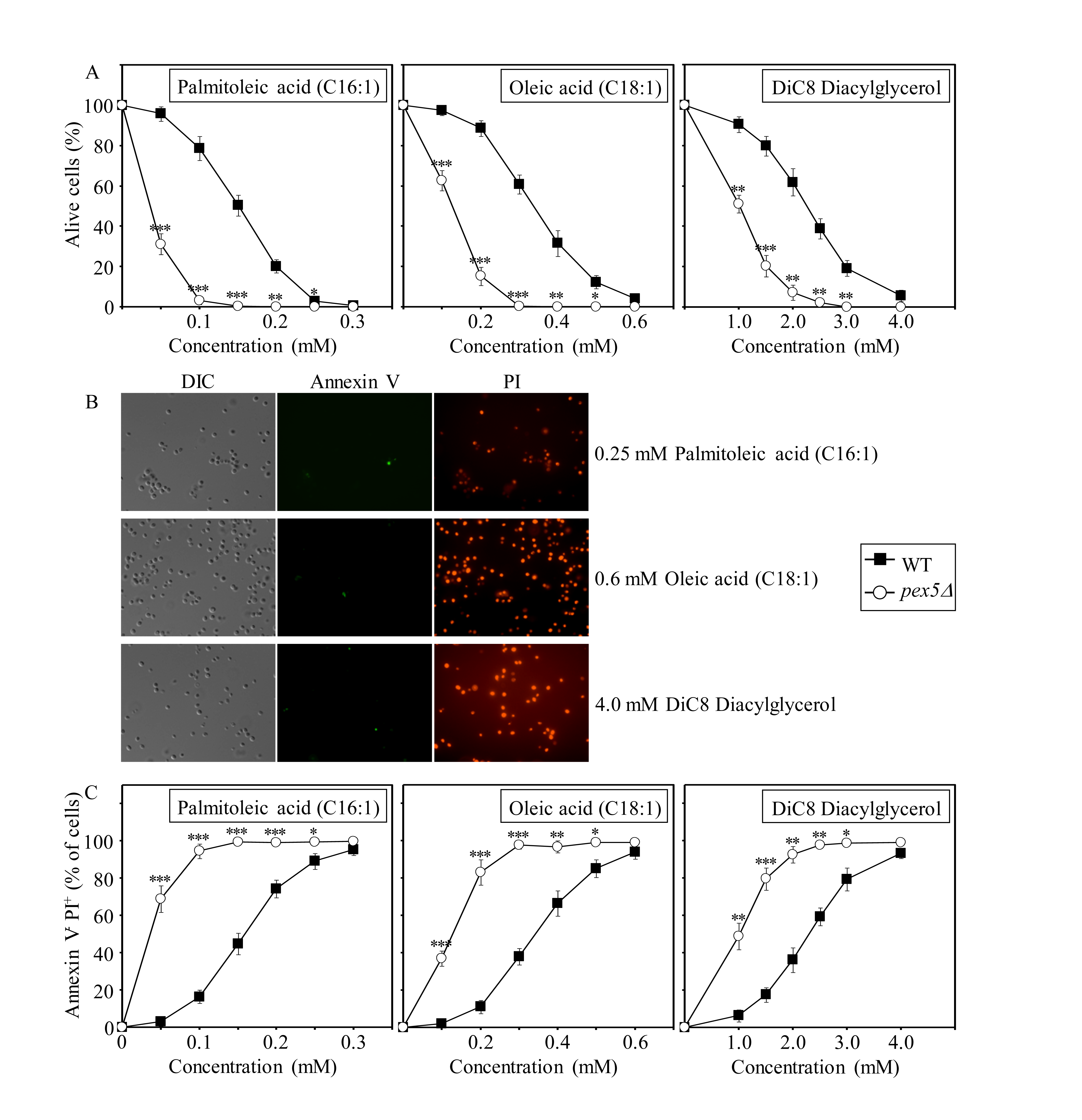

Supplement: Supplementary Figure 1 — (A) Viability of WT and pex5Δ cells treated for 2 h with palmitoleic acid, oleic acid or DiC8 diacylglycerol. (B) Fluorescence microscopy of WT yeast treated for 2 h with 0.25 mM palmitoleic acid, 0.6 mM oleic acid or 4.0 mM DiC8 diacylglycerol. Cells were co-stained with 1) Annexin V for visualizing the externalization of phosphatidylserine, a hallmark event of apoptosis; and 2) propidium iodide (PI) for visualizing the loss of plasma membrane integrity, a hallmark event of necrosis. (C) Percent of WT and pex5Δ cells that following their treatment with palmitoleic acid, oleic acid or DiC8 diacylglycerol displayed Annexin V negative and PI positive (Annexin V- and PI+) staining characteristic of necrotic cell death. Data in A and C are presented as means ± SEM (n = 3-5; ***p < 0.001; **p < 0.01; *p < 0.05). Prior to their exposure to exogenous lipids, CR yeast were grown for 2 days on 0.2% glucose. Abbreviations: DIC, differential interference contrast. [file aging-02-393-s001.tif]

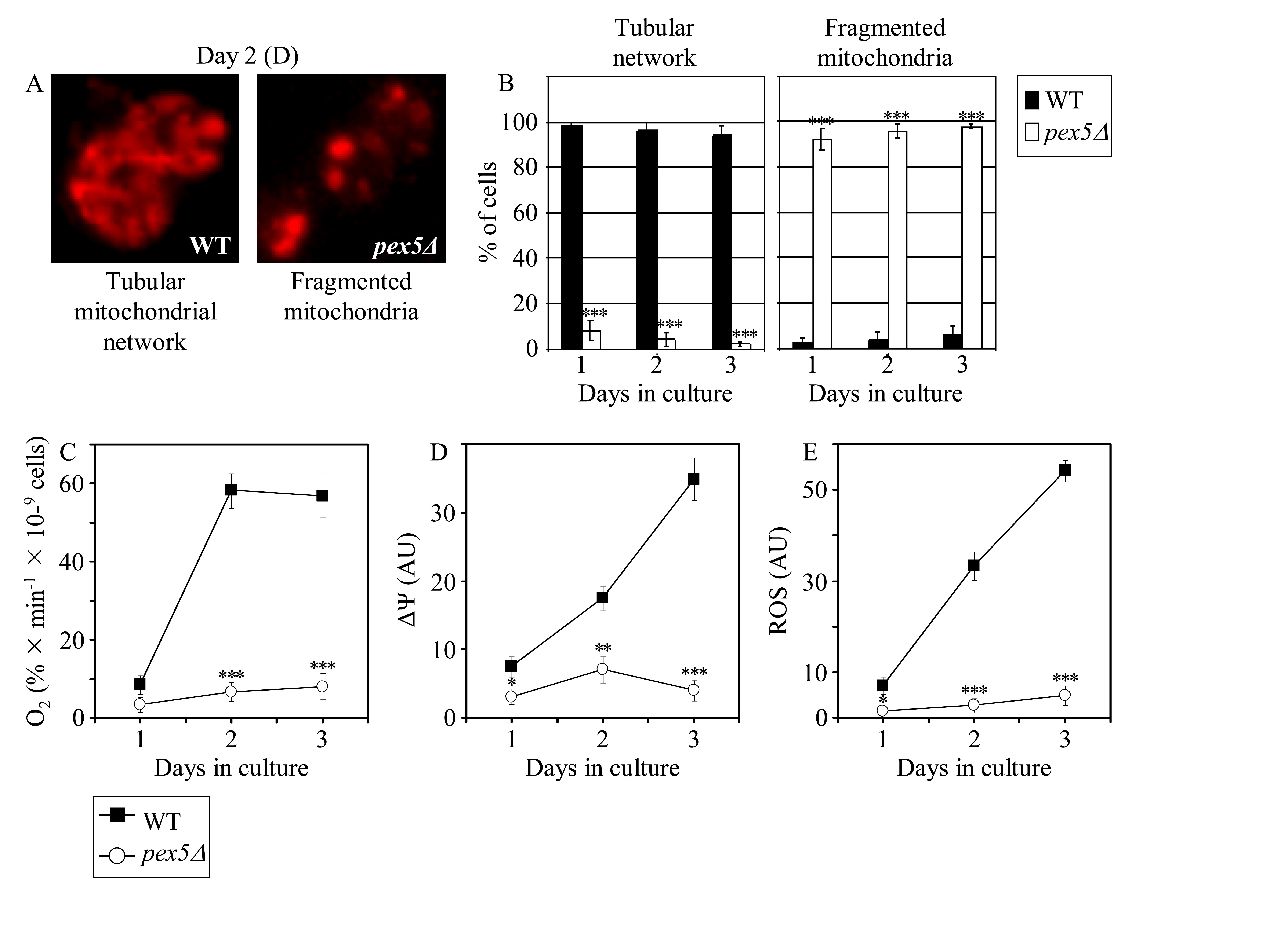

Supplement: Supplementary Figure 2 — (A) Morphology of mitochondria in WT and pex5Δ cells. Mitochondria were visualized by indirect immunofluorescence microscopy using monoclonal anti-porin primary antibodies and Alexa Fluor 568-conjugated goat anti-mouse IgG secondary antibodies. (B) Percent of WT and pex5Δ cells exhibiting a tubular mitochondrial network or fragmented mitochondria. (C - E) Oxygen consumption (C) by WT and pex5Δ cells, their mitochondrial membrane potential ΔΨ (D) and their ROS levels (E). ΔΨ and ROS were visualized in living cells by fluorescence microscopy using fluorescent dyes Rhodamine 123 or Dihydrorhodamine 123, respectively. CR yeast grown on 0.2% glucose were taken for analyses at the indicated time-points. Data in B - E are presented as means ± SEM (n = 4-15; ***p < 0.001; **p < 0.01; *p < 0.05). Abbreviation: D, diauxic growth phase. [file aging-02-393-s002.tif]

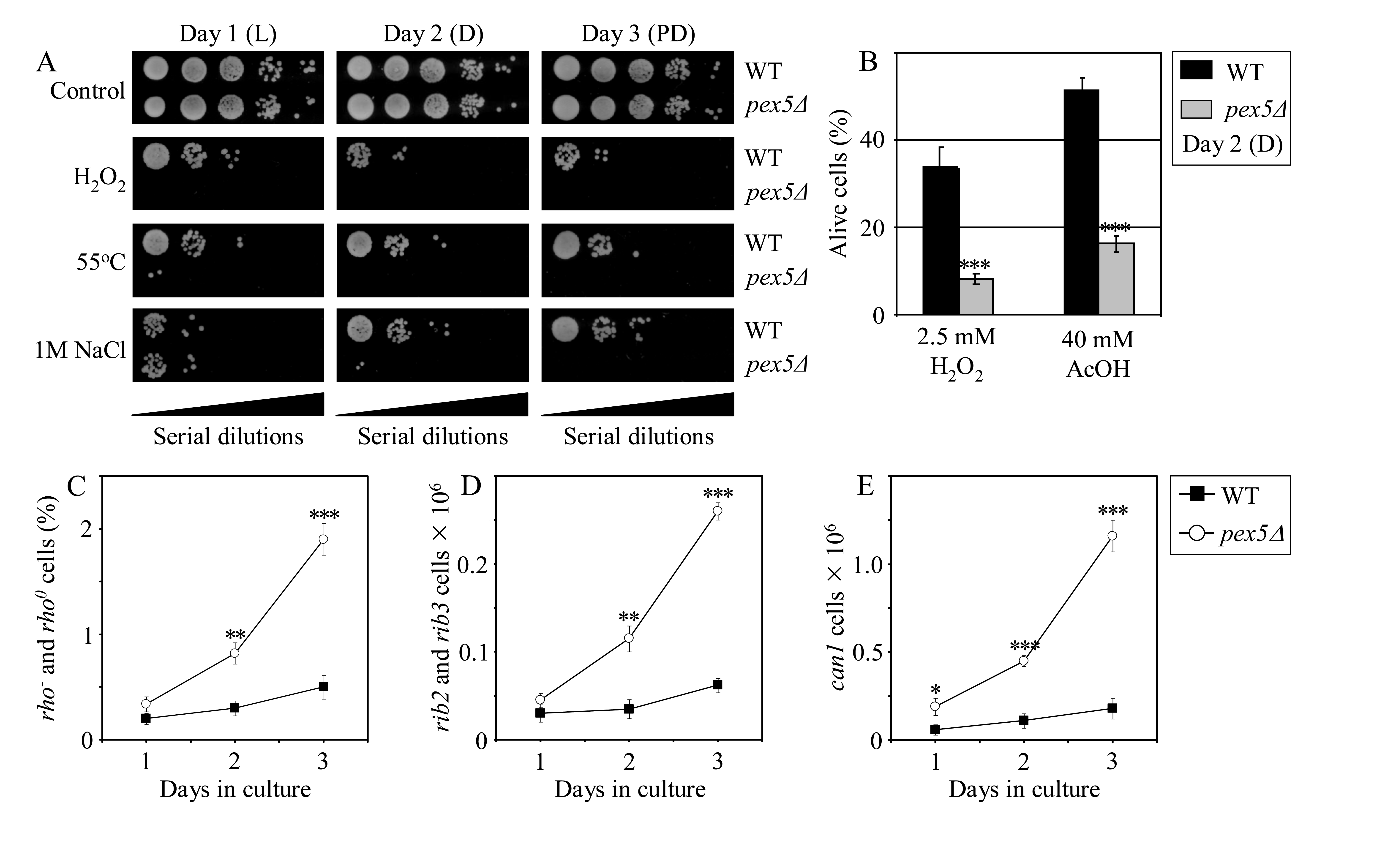

Supplement: Supplementary Figure 3 — The pex5Δ mutation reduces the resistance of CR yeast to stresses, sensitizes them to exogenously induced apoptosis and elevates the frequencies of mutations in their mitochondrial and nuclear DNA. (A) The resistance of WT and pex5Δ to chronic oxidative, thermal and osmotic stresses. (B) Viability of WT and pex5Δ cells treated for 1 h with hydrogen peroxide or acetic acid (AcOH) to induce mitochondria-controlled apoptosis. (C - E) The frequencies of rho- and rho0 deletion mutations in mitochondrial DNA (C), rib2 and rib3 point mutations in mitochondrial DNA (D), and of can1 point mutations in nuclear DNA (E) of WT and pex5Δ cells. CR yeast grown on 0.2% glucose were taken for analyses at the indicated time-points. Data in B to E are presented as means ± SEM (n = 6-9; ***p < 0.001; **p < 0.01; *p < 0.05). Abbreviations: AcOH, acetic acid; D, diauxic growth phase; L, logarithmic growth phase; PD, post-diauxic growth phase. [file aging-02-393-s003.tif]

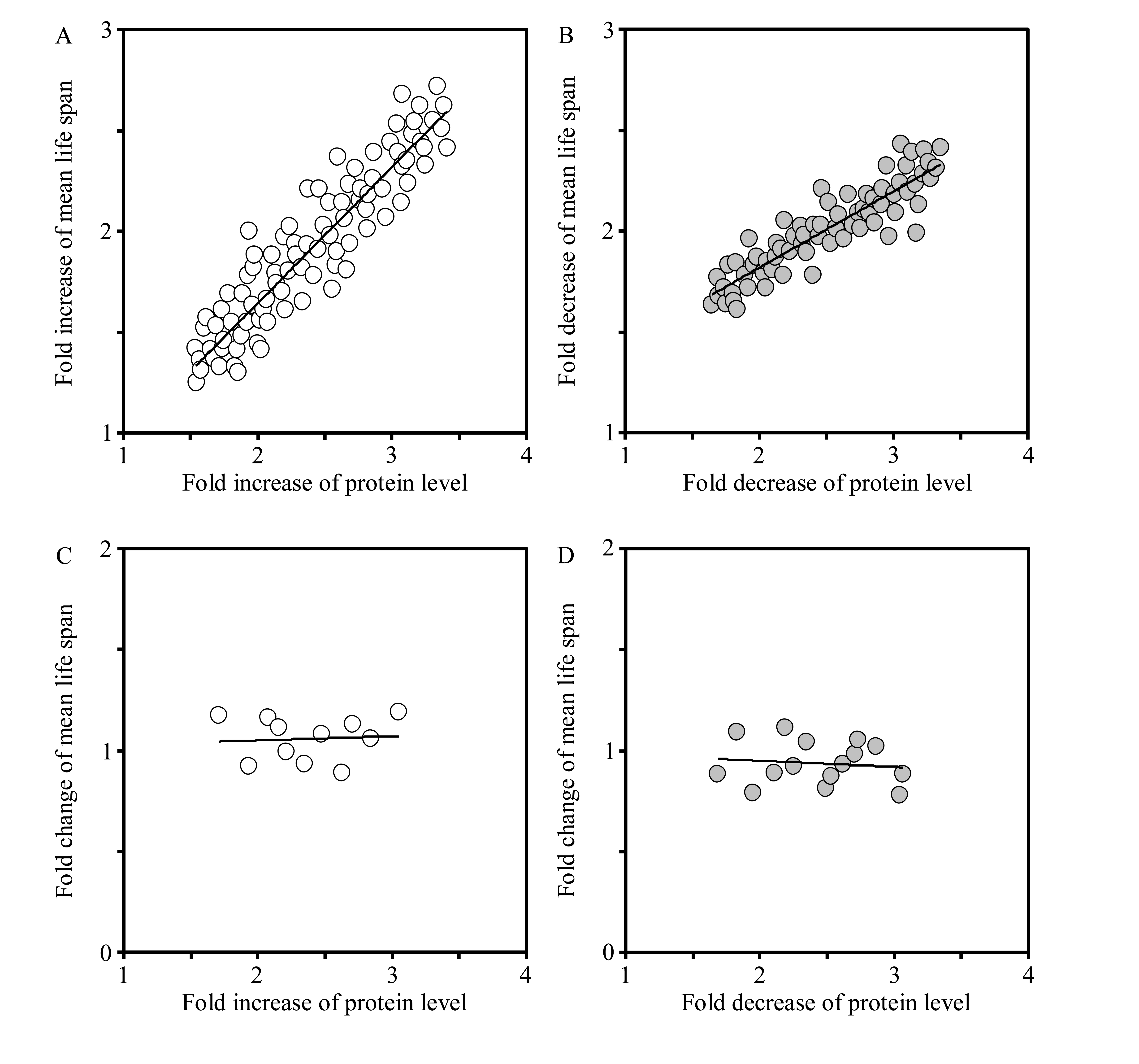

Supplement: Supplementary Figure 4 — For many proteins, the fold increase or decrease in the level of a protein enriched or depleted in pex5Δ yeast under CR correlates with the fold increase or decrease (respectively) in the mean CLS of a mutant strain lacking it. Plots comparing the fold increase or decrease in the levels of proteins enriched or depleted in pex5Δ yeast when calorie supply is limited and the fold increase or decrease (respectively) in the mean CLS of the single-gene-deletion mutant strains that lack these proteins and grow under CR. Each point shows the data for a single protein and a mutant strain that lacks it. Data are presented only for proteins whose levels were increased or decreased by more than 50% in pex5Δ cells (as compared to WT cells) grown under CR on 0.2% glucose. Linear regression is shown. [file aging-02-393-s004.tif]

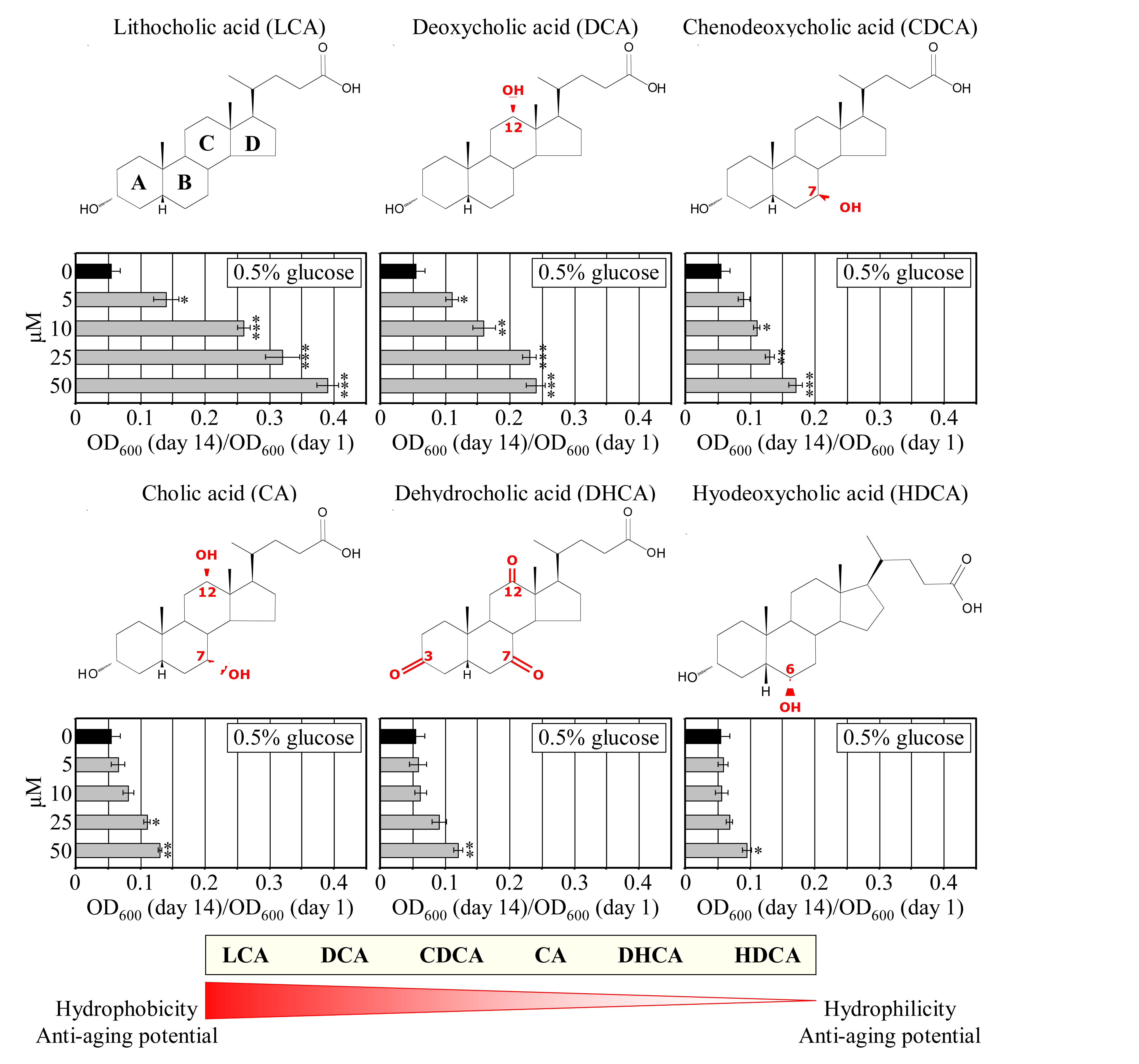

Supplement: Supplementary Figure 5 — In the microplate assay, lithocholic acid - the most hydrophobic bile acid species - displays the greatest ability to extend the CLS of the short-lived pex5Δ mutant strain under CR conditions. The effect of various concentrations of different bile acids on the CLS of the short-lived pex5Δ mutant strain grown under CR on 0.5% glucose is shown. The "OD600 at day 14/OD600 at day 1" ratio was used as a measure of CLS. Data are presented as means ± SEM (n = 3-5; ***p < 0.001; **p < 0.01; *p < 0.05). [file aging-02-393-s005.tif]

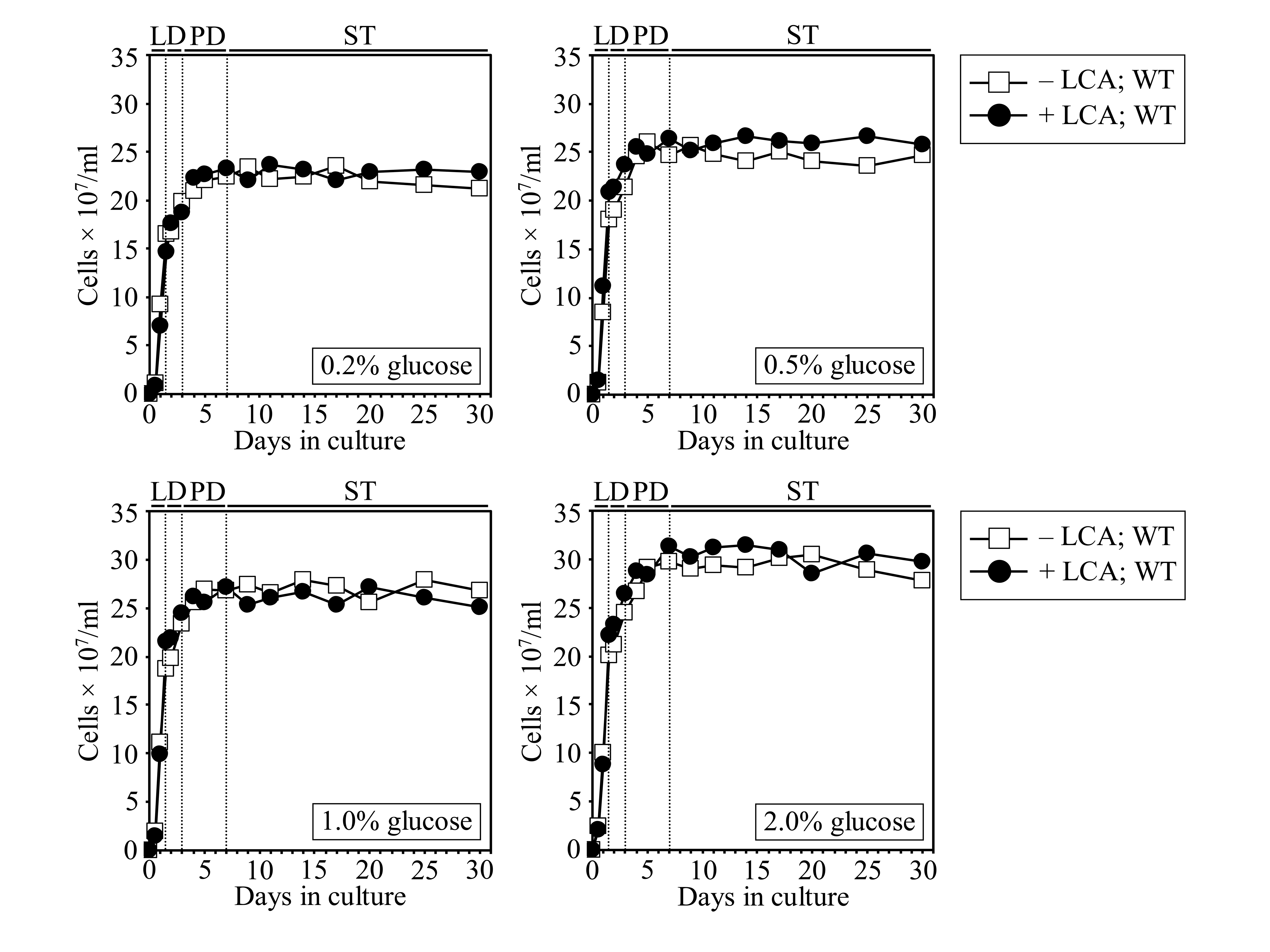

Supplement: Supplementary Figure 6 — Kinetics of growth for WT strain in medium initially containing 0.2%, 0.5%, 1.0% or 2.0% glucose in the presence of LCA (50 μM ) or in its presence. Each plot shows a representative experiment repeated 4-7 times in triplicate with similar results. Abbreviations: D, diauxic growth phase; L, logarithmic growth phase; PD, post-diauxic growth phase; ST, stationary growth phase. [file aging-02-393-s006.tif]

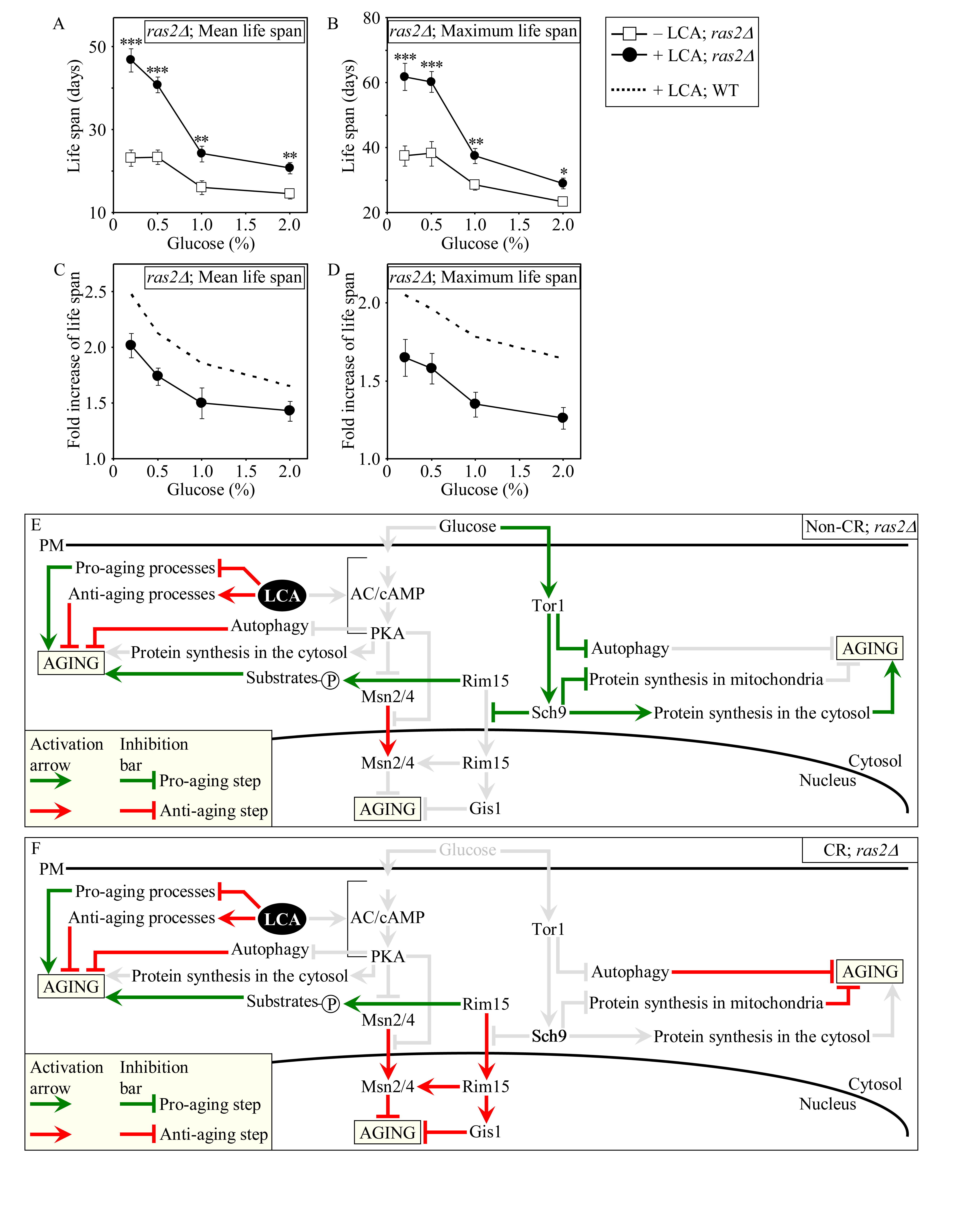

Supplement: Supplementary Figure 7 — (A and B) Effect of LCA on the mean (A) and maximum (B) life spans of chronologically aging ras2Δ strain. Data are presented as means ± SEM (n = 4-7; ***p < 0.001; **p < 0.01; *p < 0.05). (C and D) Effect of LCA on the fold increase in the mean (C) or maximum (D) life spans of chronologically aging ras2Δ and WT strains. Data are presented as means ± SEM (n = 4-7). Cells in A to D were cultured in medium initially containing 0.2%, 0.5%, 1% or 2% glucose in the presence of LCA (50 μM) or in its absence. Chronological survival data for ras2Δ strain are provided in Supplementary Figure 11. (E and F) Outline of pro- and anti-aging processes that are controlled by the TOR and/or cAMP/PKA signaling pathways and are modulated by LCA in ras2Δ cells grown under non-CR (E) or CR (F) conditions. Activation arrows and inhibition bars denote pro-aging processes (displayed in green color) or anti-aging processes (displayed in red color). Abbreviations: PM, plasma membrane. [file aging-02-393-s007.tif]

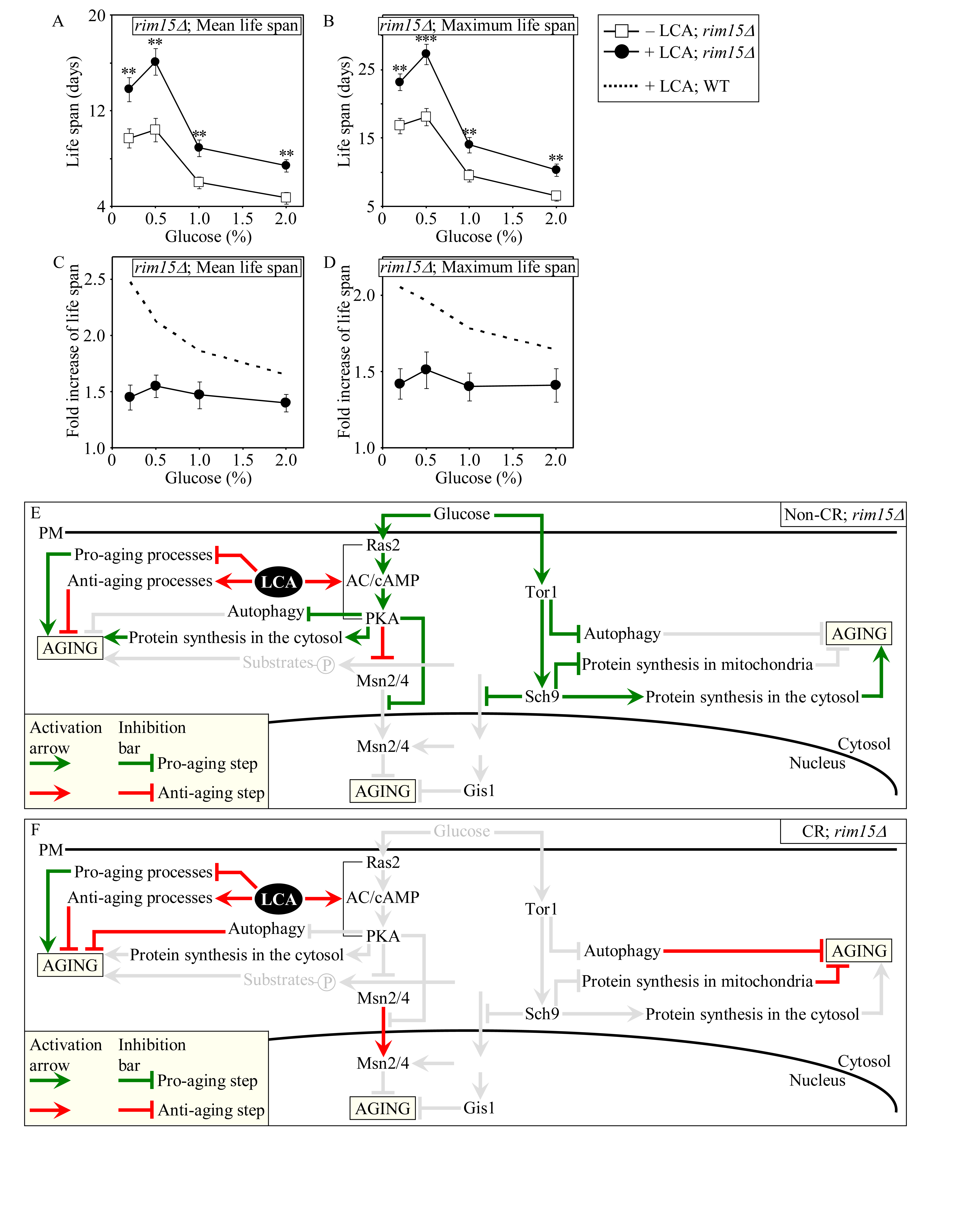

Supplement: Supplementary Figure 8 — (A and B) Effect of LCA on the mean (A) and maximum (B) life spans of chronologically aging rim15Δ strain. Data are presented as means ± SEM (n = 5-7; ***p < 0.001; **p < 0.01). (C and D) Effect of LCA on the fold increase in the mean (C) or maximum (D) life spans of chronologically aging rim15Δ and WT strains. Data are presented as means ± SEM (n = 5-7). Cells in A to D were cultured in medium initially containing 0.2%, 0.5%, 1% or 2% glucose in the presence of LCA (50 μM) or in its absence. Chronological survival data for rim15Δ strain are provided in Supplementary Figure 12. (E and F) Outline of pro- and anti-aging processes that are controlled by the TOR and/or cAMP/PKA signaling pathways and are modulated by LCA in rim15Δ cells grown under non-CR (E) or CR (F) conditions. Activation arrows and inhibition bars denote pro-aging processes (displayed in green color) or anti-aging processes (displayed in red color). Abbreviations: PM, plasma membrane. [file aging-02-393-s008.tif]

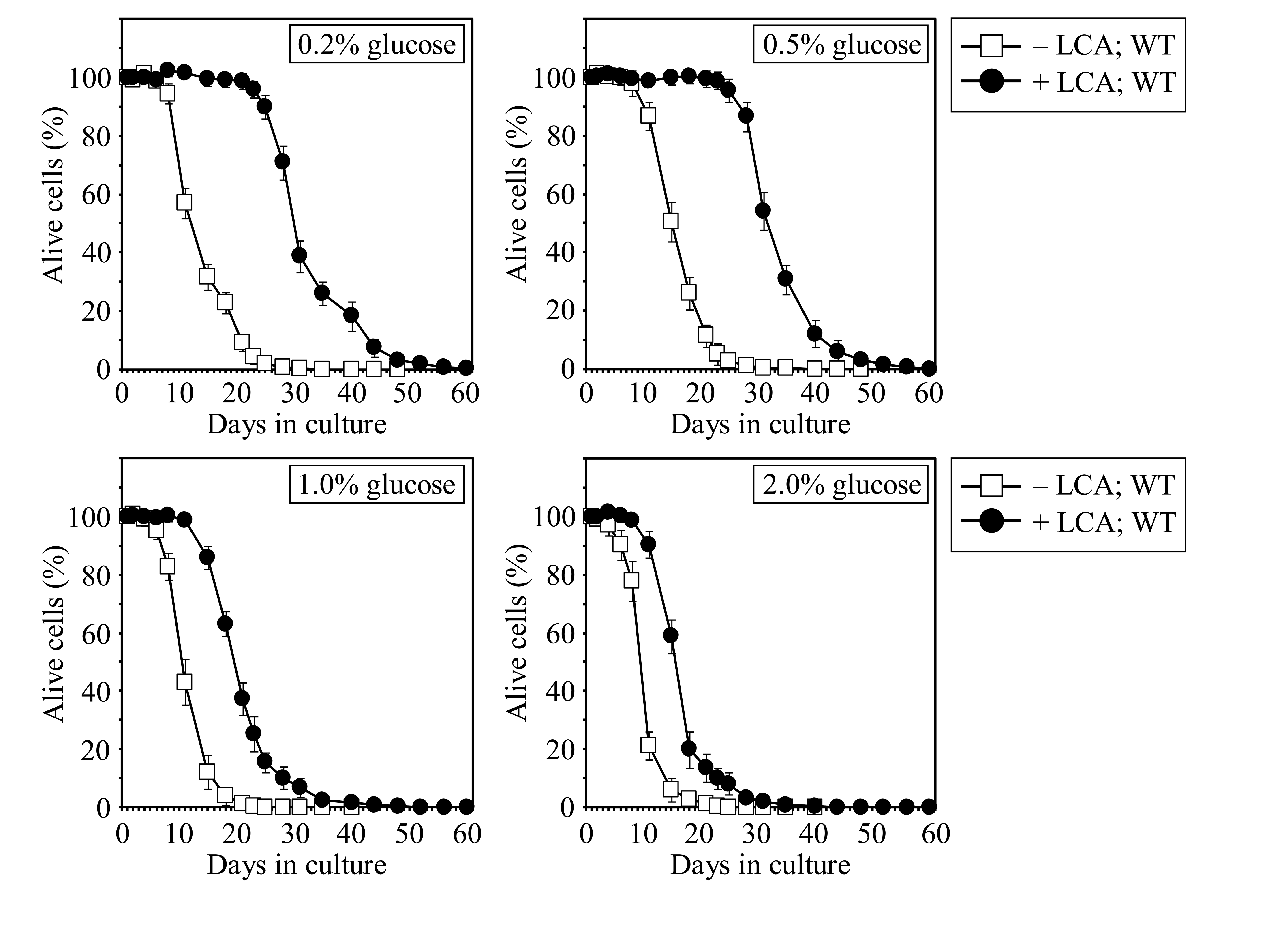

Supplement: Supplementary Figure 9 — Chronological survival data for WT strain cultured in medium initially containing 0.2%, 0.5%, 1% or 2% glucose in the presence of LCA (50 μM) or in its absence. Dataset for Figure 8. [file aging-02-393-s009.tif]

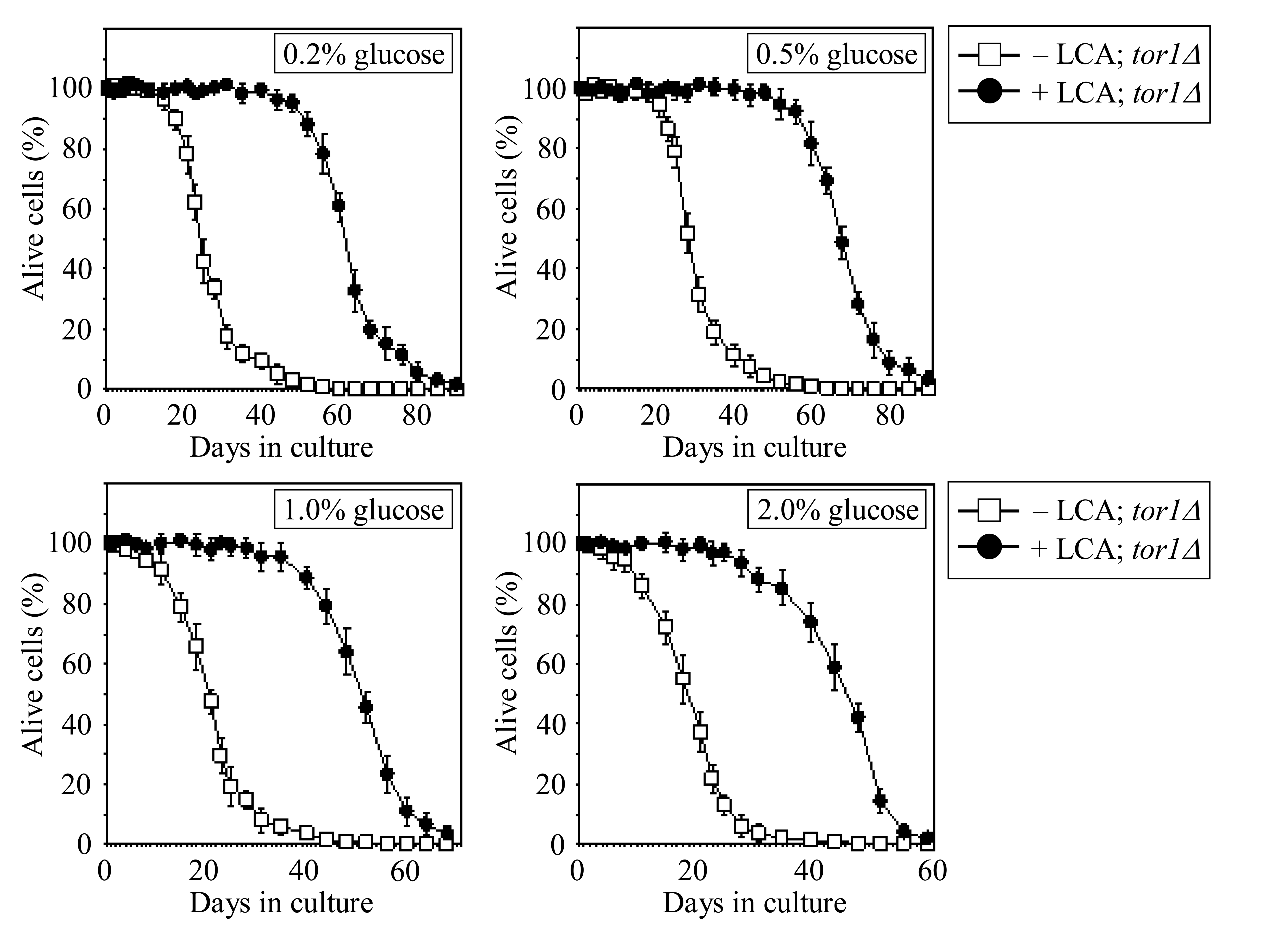

Supplement: Supplementary Figure 10 — Chronological survival data for tor1Δ strain cultured in medium initially containing 0.2%, 0.5%, 1% or 2% glucose in the presence of LCA (50 μM) or in its absence. Dataset for Figure 9. [file aging-02-393-s010.tif]

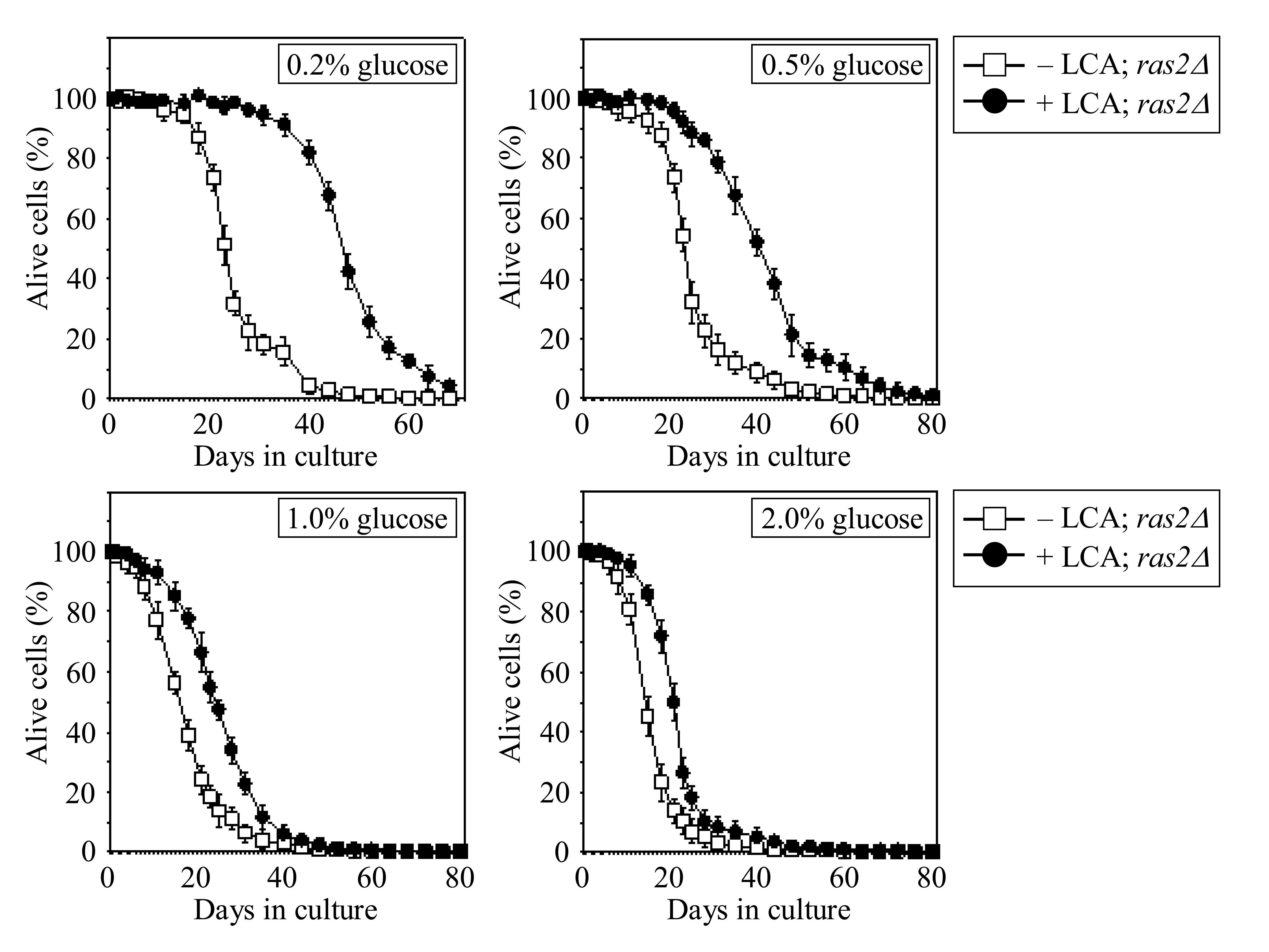

Supplement: Supplementary Figure 11 — Chronological survival data for ras2Δ strain cultured in medium initially containing 0.2%, 0.5%, 1% or 2% glucose in the presence of LCA (50 μM) or in its absence. Dataset for Supplementary Figure 7. [file aging-02-393-s011.tif]

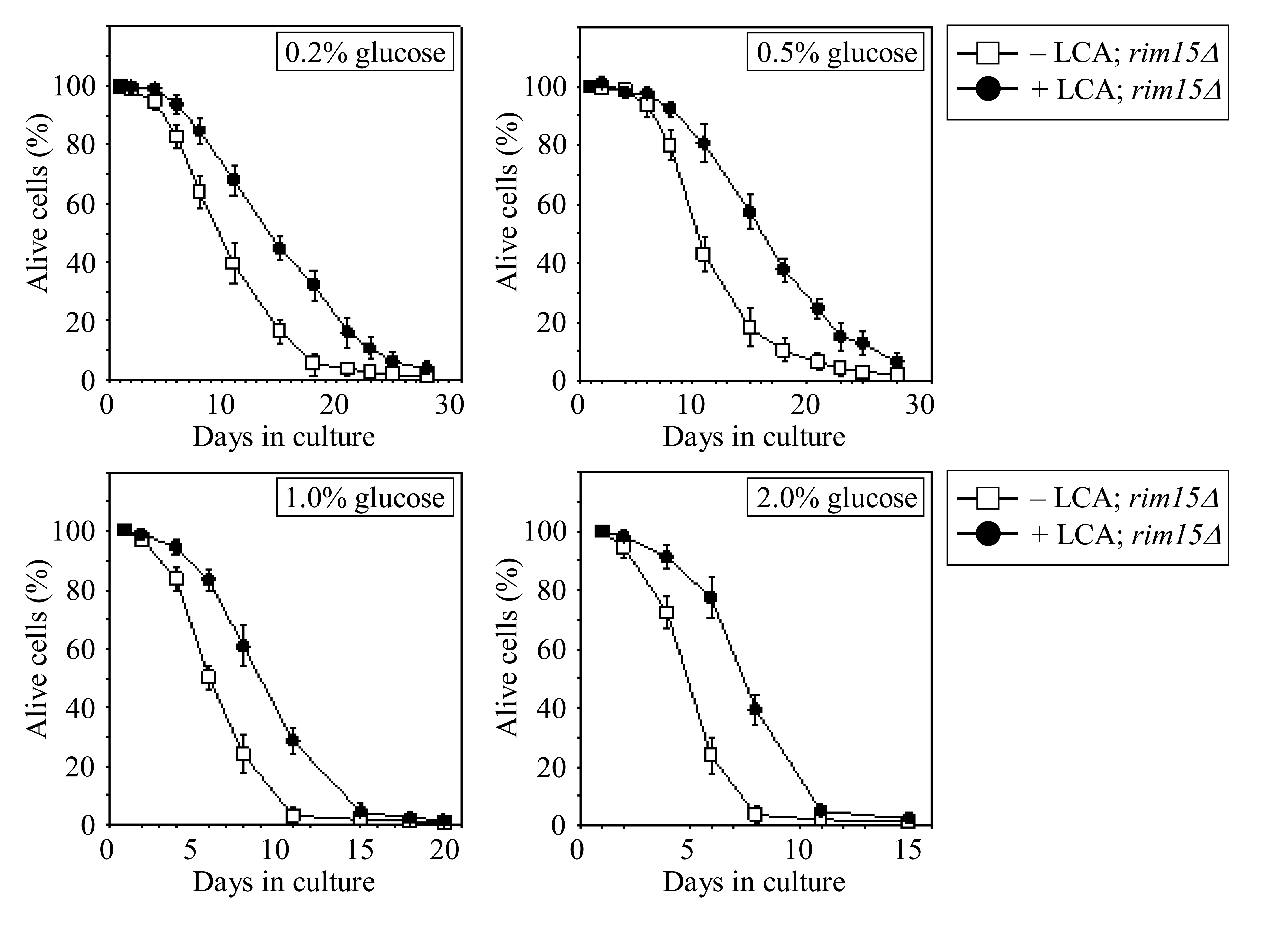

Supplement: Supplementary Figure 12 — Chronological survival data for rim15Δ strain cultured in medium initially containing 0.2%, 0.5%, 1% or 2% glucose in the presence of LCA (50 μM) or in its absence. Dataset for Supplementary Figure 8. [file aging-02-393-s012.tif]
