# Supplementary material for: Chemical genetic screen identifies lithocholic acid as an anti-aging compound that extends yeast chronological life span in a TOR-independent manner, by modulating housekeeping longevity assurance processes
Source: Aging (Albany NY). 2010 Jul 7;2(7):393–414. doi: 10.18632/aging.100168 (PMC2933888; doi:10.18632/aging.100168)
Supplement: Supplementary Table 1 [file aging-02-393-s001.pdf]

Table S1. Known anti-aging compounds, their abilities to increase life span in different organisms (under caloric or dietary restriction [CR or DR, respectively], on a standard diet or fed a high-calorie diet), and the mechanisms of their anti-aging action.

Compound	Increases life span*		Mechanism
	CR/DR	Standard or high-calorie diet	
Caffeine	NT	+ (yeast, CLS) [1]	By inhibiting TORC1, modulates unspecified longevity-related processes [1] governed by the TOR pathway [2,3]
Li^+	+ (nematodes**) [4]	+ (nematodes) [4]	By altering transcription of genes involved in histone methylation, nucleosome composition and chromatin structure, modulates unspecified longevity-related processes [4] known to be influenced by age-related chromatin reorganization [5]
Lipoic acid, propyl gallate, trolox and taxifolin	NT	+ (nematodes [6] (all); fruit flies [7] (lipoic acid))	Antioxidants that may increase life span by detoxifying free radicals and/or enhancing resistance to age-related oxidative stress [6,7]
Metformin, buformin and phenformin	- (nematodes**) [8]	+ (nematodes [8]; mice [9])	Type 2 diabetes therapeutics that - by activating LKB1/AMPK signaling and thereby inhibiting TORC1 [10,11] - modulate unspecified longevity-related processes [8,9] known to be governed by the TOR pathway [2,3,11]
Methionine sulfoximine	NT	+ (yeast, CLS) [12]	By inhibiting glutamine synthetase and reducing both intracellular glutamine level and TORC1-signaling [13], increases life span - perhaps by activating gluconeogenesis and enhancing stress resistance [12]
Mianserin	- (nematodes) [14]	+ (nematodes) [14]	A serotonin receptor antagonist used as an antidepressant in humans; may increase life span by inhibiting neurotransmission related to food sensing, thereby mimicking a DR-like physiological state [14]***
Rapamycin	+ (fruit flies) [16]	+ (yeast, CLS [1,12,17] and RLS [18]; fruit flies [16]; mice [19]; rodent fibroblasts,	By inhibiting TORC1 (yeast and fruit flies) [1,16,17,21] and mTORC1 (mammals)

		human epithelium and fibrosarcoma cells - all RLS [20])	[11,19,21], increases life span by activating macroautophagy (yeast and fruit flies) [16,22] and inhibiting cap-dependent protein translation (fruit flies and mice) [16,19] as well as - perhaps - by promoting gluconeogenesis (yeast) [12], enhancing stress resistance (yeast) [12,18], and increasing neutral lipid levels (fruit flies) [16]
Resveratrol	- (yeast, RLS [23]; fruit flies [24]; mice [25])	+ (yeast, RLS [23] but not CLS [23]; nematodes [24]; fruit flies [24]; fishes [26]; mice [25,28]; human fibroblasts, RLS [27])****	Increases life span by modulating a number of longevity-related processes (*e.g.*, by altering transcription of numerous genes involved in key longevity pathways, stimulating p53 deacetylation, increasing insulin sensitivity and mitochondrial number, reducing IGF-1 levels, activating AMPK and PGC-1α, promoting ER stress response, repressing transcription of PPAR-γ, inhibiting adipocyte differentiation, accelerating storage fat mobilization, inhibiting mTORC1, and activating autophagy [23,25,27-35); its life-extending ability in yeast, nematodes and fruit flies depends on Sir2p - a member of the conserved sirtuin family of NAD^+-dependent protein deacetylases/mono-ADP-ribosyltransferases [23,24,29]*****
SkQ1	NT	+ (fungi, daphnias, fruit flies, mice) [43]	By being specifically targeted to mitochondria, acts as an antioxidant that may increase life span by preventing oxidative damage to proteins and lipids (*i.e.*, cardiolipin), altering mitochondrial morphology, reducing hydrogen peroxide-induced apoptosis and necrosis, and/or slowing down the age-related phosphorylation of histone H2AX [43]
Sodium nitroprusside	NT	+ (human PBMC,RLS) [44]	By activating expression of the human sirtuin SIRT1 and thereby increasing the extent

			of SIRT1-dependent histone H4 lysine 16 deacetylation, may cause the development of an anti-aging pattern of transcription of numerous genes involved in longevity regulation [44]
Spermidine	NT	+ (yeast, CLS; nematodes; fruit flies; human PBMC, RLS) [45]	By inhibiting histone acetyltransferases and promoting histone H3 deacetylation, increases life span by activating transcription of numerous autophagy-related genes; the resulting induction of autophagy suppresses age-related necrotic cell death [45]
Valproic acid	NT	+ (nematodes) [46]	Is used as a mood stabilizer and an anticonvulsant in humans; may increase life span by promoting nuclear localization of the DAF-16 forkhead transcription factor, thereby reducing the pro-aging effect of the insulin/IGF-1 signaling pathway [46]
LY294002	NT	+ (human fibrosarcoma cells, RLS) [47]	An inhibitor of phosphatidylinositol-3-kinase that – by reducing mTORC1 signaling [2,3] – modulates unspecified longevity-related processes [47] known to be governed by the TOR pathway [2,3,11]
U0126	NT	+ (human fibrosarcoma cells, RLS) [47]	An inhibitor of the protein kinase MEK that – by reducing mTORC1 signaling [2,3] – modulates unspecified longevity-related processes [47] known to be governed by the TOR pathway [2,3,11]

[*] Mean, median and/or maximum life spans.

[**] Nematodes carrying mutations that mimic DR under non-DR conditions [4,8].

[***] The ability of mianserin to increase nematode life span can only be seen in liquid media [14], whereas in solid media the compound reduces life span [15].

[****] Increases the replicative life span of yeast grown under non-CR conditions [23] only in one out of four different yeast strain backgrounds [36]; one group has been unable to reproduce the life span extension by resveratrol in nematodes and fruit flies [37]; increases the life of mice only if fed a high-calorie diet, but not a standard diet [25,28].

***** Although the life-extending ability of resveratrol in yeast, nematodes and fruit flies depends on Sir2p [23,24,29], it is currently debated whether this anti-aging compound binds to Sir2p (or SIRT1, a mammalian sirtuin) in vivo and/or activates Sir2p or SIRT1 in living cells [30,36-40]; importantly, resveratrol has been shown to inhibit or activate many proteins other than sirtuins by interacting with them [41,42].

Abbreviations: AMPK, the AMP-activated serine/threonine protein kinase; CLS, chronological life span; IGF-1, insulin-like growth factor 1; LKB1, a serine/threonine protein kinase that phosphorylates and activates AMPK; mTORC1, the mammalian target of rapamycin complex 1; NT, not tested; PBMC, peripheral blood mononuclear cells; PGC-1α, peroxisome proliferator-activated receptor-γ co-activator 1α; RLS, replicative life span; TORC1, the yeast target of rapamycin complex 1.

References for Table S1

1. Wanke V, Cameroni E, Uotila A, Piccolis M, Urban J, Loewith R, De Virgilio C. Caffeine extends yeast lifespan by targeting TORC1. Mol Microbiol. 2008; 69: 277-285.

2. Wullschleger S, Loewith R, Hall MN. TOR signaling in growth and metabolism. Cell 2006; 124: 471-484.

3. Laplante M, Sabatini DM. mTOR signaling at a glance. J Cell Sci. 2009; 122: 3589-3594.

4. McColl G, Killilea DW, Hubbard AE, Vantipalli MC, Melov S, Lithgow GJ. Pharmacogenetic analysis of lithium-induced delayed aging in Caenorhabditis elegans. J Biol Chem. 2008; 283: 350-357.

5. Sinclair DA, Oberdoerffer P. The ageing epigenome: damaged beyond repair? Ageing Res Rev. 2009; 8: 189-198.

6. Benedetti MG, Foster AL, Vantipalli MC, White MP, Sampayo JN, Gill MS, Olsen A, Lithgow GJ. Compounds that confer thermal stress resistance and extended lifespan. Exp Gerontol. 2008; 43: 882-891.

7. Bauer JH, Goupil S, Garber GB, Helfand SL. An accelerated assay for the identification of lifespan-extending interventions in Drosophila melanogaster. Proc Natl Acad Sci USA 2004; 101: 12980-12985.

8. Onken B, Driscoll M. Metformin induces a dietary restriction-like state and the oxidative stress response to extend C. elegans healthspan via AMPK, LKB1, and SKN-1. PLoS ONE 2010; 5: e8758.

9. Anisimov VN, Berstein LM, Egormin PA, Piskunova TS, Popovich IG, Zabezhinski MA, Tyndyk ML, Yurova MV, Kovalenko IG, Poroshina TE, Semenchenko AV. Anisimov VN, Berstein LM, Egormin PA, Piskunova TS, Popovich IG, Zabezhinski MA, Tyndyk ML, Yurova MV, Kovalenko IG, Poroshina TE, Semenchenko AV. Metformin slows down aging and extends life span of female SHR mice. Cell Cycle 2008; 7: 2769-2773.

10. Shaw RJ, Lamia KA, Vasquez D, Koo SH, Bardeesy N, Depinho RA, Montminy M, Cantley LC. The kinase LKB1 mediates glucose homeostasis in liver and therapeutic effects of metformin. Science 2005; 310: 1642-1646.

11. Shaw RJ. LKB1 and AMP-activated protein kinase control of mTOR signalling and growth. Acta Physiol. 2009; 196: 65-80.

12. Powers RW 3rd, Kaeberlein M, Caldwell SD, Kennedy BK, Fields S. Extension of chronological life span in yeast by decreased TOR pathway signaling. Genes Dev. 2006; 20: 174-184.

13. Crespo JL, Powers T, Fowler B, Hall MN. The TOR-controlled transcription activators GLN3, RTG1, and RTG3 are regulated in response to intracellular levels of glutamine. Proc. Natl. Acad. Sci. USA 2002; 99: 6784-6789.

14. Petrascheck M, Ye X, Buck LB. An antidepressant that extends lifespan in adult Caenorhabditis elegans. Nature 2007; 450: 553-556.

15. Zarse K, Ristow M. Antidepressants of the serotonin-antagonist type increase body fat and decrease lifespan of adult Caenorhabditis elegans. PLoS ONE 2008; 3: e4062.

16. Bjedov I, Toivonen JM, Kerr F, Slack C, Jacobson J, Foley A, Partridge L. Mechanisms of life span extension by rapamycin in the fruit fly Drosophila melanogaster. Cell Metab. 2010; 11: 35-46.

17. Bonawitz ND, Chatenay-Lapointe M, Pan Y, Shadel GS. Reduced TOR signaling extends chronological life span via increased respiration and upregulation of mitochondrial gene expression. Cell Metab. 2007; 5: 265-277.

18. Medvedik O, Lamming DW, Kim KD, Sinclair DA. MSN2 and MSN4 link calorie restriction and TOR to sirtuin-mediated lifespan extension in Saccharomyces cerevisiae. PLoS Biol. 2007; 5: e261.

19. Harrison DE, Strong R, Sharp ZD, Nelson JF, Astle CM, Flurkey K, Nadon NL, Wilkinson JE, Frenkel K, Carter CS, Pahor M, Javors MA, Fernandez E, Miller RA. Rapamycin fed late in life extends lifespan in genetically heterogeneous mice. Nature 2009; 460: 392-395.

20. Demidenko ZN, Zubova SG, Bukreeva EI, Pospelov VA, Pospelova TV, Blagosklonny MV. Rapamycin decelerates cellular senescence. Cell Cycle 2009; 8: 1888-1895.

21. Wullschleger S, Loewith R, Hall MN. TOR signaling in growth and metabolism. Cell 2006; 124: 471-484.

22. Alvers AL, Wood MS, Hu D, Kaywell AC, Dunn WA Jr, Aris JP. Autophagy is required for extension of yeast chronological life span by rapamycin. Autophagy 2009; 5: 847-849.

23. Howitz KT, Bitterman KJ, Cohen HY, Lamming DW, Lavu S, Wood JG, Zipkin RE, Chung P, Kisielewski A, Zhang LL, Scherer B, Sinclair DA. Small molecule activators of sirtuins extend Saccharomyces cerevisiae lifespan. Nature 2003; 425: 191-196.

24. Wood JG, Rogina B, Lavu S, Howitz K, Helfand SL, Tatar M, Sinclair D. Sirtuin activators mimic caloric restriction and delay ageing in metazoans. Nature 2004; 430: 686-689.

25. Baur JA, Pearson KJ, Price NL, Jamieson HA, Lerin C, Kalra A, Prabhu VV, Allard JS, Lopez-Lluch G, Lewis K, Pistell PJ, Poosala S, Becker KG, Boss O, Gwinn D, Wang M, Ramaswamy S, Fishbein KW, Spencer RG, Lakatta EG, Le Couteur D, Shaw RJ, Navas P, Puigserver P, Ingram DK, de Cabo R, Sinclair DA. Resveratrol improves health and survival of mice on a high-calorie diet. Nature 2006; 444: 337-342.

26. Valenzano DR, Terzibasi E, Genade T, Cattaneo A, Domenici L, Cellerino A. Resveratrol prolongs lifespan and retards the onset of age-related markers in a short-lived vertebrate. Curr Biol. 2006; 16: 296-300.

27. Demidenko ZN, Blagosklonny MV. At concentrations that inhibit mTOR, resveratrol suppresses cellular senescence. Cell Cycle 2009; 8: 1901-1904.

28. Pearson KJ, Baur JA, Lewis KN, Peshkin L, Price NL, Labinskyy N, Swindell WR, Kamara D, Minor RK, Perez E, Jamieson HA, Zhang Y, Dunn SR, Sharma K, Pleshko N, Woollett LA, Csiszar A, Ikeno Y, Le Couteur D, Elliott PJ, Becker KG, Navas P, Ingram DK, Wolf NS, Ungvari Z, Sinclair DA, de Cabo R. Resveratrol delays age-related deterioration and mimics transcriptional aspects of dietary restriction without extending life span. Cell Metab. 2008; 8: 157-168.

29. Viswanathan M, Kim SK, Berdichevsky A, Guarente L. A role for SIR-2.1 regulation of ER stress response genes in determining C. elegans life span. Dev. Cell 2005; 9: 605-615.

30. Lagouge M, Argmann C, Gerhart-Hines Z, Meziane H, Lerin C, Daussin F, Messadeq N, Milne J, Lambert P, Elliott P, Geny B, Laakso M, Puigserver P, Auwerx J. Resveratrol improves mitochondrial function and protects against metabolic disease by activating SIRT1 and PGC-1α. Cell 2006; 127: 1109-1122.

31. Picard F, Kurtev M, Chung N, Topark-Ngarm A, Senawong T, Machado De Oliveira R, Leid M, McBurney MW, Guarente L (2004) Sirt1 promotes fat mobilization in white adipocytes by repressing PPAR-γ. Nature 2004; 429: 771-776.

32. Blagosklonny MV. TOR-driven aging: speeding car without brakes. Cell Cycle 2009; 8: 4055-4059.

33. Morselli E, Maiuri MC, Markaki M, Megalou E, Pasparaki A, Palikaras K, Criollo A, Galluzzi L, Malik SA, Vitale I, Michaud M, Madeo F, Tavernarakis N, Kroemer G. Caloric restriction and resveratrol promote longevity through the Sirtuin-1-dependent induction of autophagy. Cell Death Dis. 2010; 1: e10; doi:10.1038/cddis.2009.8.

34. Morselli E, Maiuri MC, Markaki M, Megalou E, Pasparaki A, Palikaras K, Criollo A, Galluzzi L, Malik SA, Vitale I, Michaud M, Madeo F, Tavernarakis N, Kroemer G. The life span-prolonging effect of sirtuin-1 is mediated by autophagy. Autophagy 2010; 6: 186-188.

35. Morselli E, Galluzzi L, Kepp O, Criollo A, Maiuri MC, Tavernarakis N, Madeo F, Kroemer G. Autophagy mediates pharmacological lifespan extension by spermidine and resveratrol. Aging 2009; 1: 961-970.

36. Kaeberlein M, McDonagh T, Heltweg B, Hixon J, Westman EA, Caldwell SD, Napper A, Curtis R, DiStefano PS, Fields S, Bedalov A, Kennedy BK. Substrate-specific activation of sirtuins by resveratrol. J Biol Chem. 2005; 280: 17038-17045.

37. Bass TM, Weinkove D, Houthoofd K, Gems D, Partridge L. Effects of resveratrol on lifespan in Drosophila melanogaster and Caenorhabditis elegans. Mech. Ageing Dev. 2007; 128: 546-552.

38. Borra MT, Smith BC, Denu JM. Mechanism of human SIRT1 activation by resveratrol. J Biol Chem. 2005; 280: 17187-17195.

39. Denu JM. The Sir 2 family of protein deacetylases. Curr Opin Chem Biol. 2005; 9: 431-440.

40. Kaeberlein M, Kennedy BK. Does resveratrol activate yeast Sir2 in vivo? Aging Cell 2007; 6: 415-416.

41. Harikumar KB, Aggarwal BB. Resveratrol: a multitargeted agent for age-associated chronic diseases. Cell Cycle 2008; 7: 1020-1035.

42. Shakibaei M, Harikumar KB, Aggarwal BB. Resveratrol addiction: to die or not to die. Mol Nutr Food Res. 2009; 53: 115-128.

43. Skulachev VP, Anisimov VN, Antonenko YN, Bakeeva LE, Chernyak BV, Erichev VP, Filenko OF, Kalinina NI, Kapelko VI, Kolosova NG, Kopnin BP, Korshunova GA, Lichinitser MR, Obukhova LA, Pasyukova EG, Pisarenko OI, Roginsky VA, Ruuge EK, Senin II, Severina II, Skulachev MV, Spivak IM, Tashlitsky VN, Tkachuk VA, Vyssokikh MY, Yaguzhinsky LS, Zorov DB. An attempt to prevent senescence: a mitochondrial approach. Biochim Biophys Acta 2009; 1787: 437-461.

44. Engel N, Mahlknecht U. Aging and anti-aging: unexpected side effects of everyday medication through sirtuin1 modulation. Int. J. Mol. Med. 2008; 21: 223-232.

45. Eisenberg T, Knauer H, Schauer A, Büttner S, Ruckenstuhl C, Carmona-Gutierrez D, Ring J, Schroeder S, Magnes C, Antonacci L, Fussi H, Deszcz L, Hartl R, Schraml E, Criollo A, Megalou E, Weiskopf D, Laun P, Heeren G, Breitenbach M, Grubeck-Loebenstein B, Herker E, Fahrenkrog B, Fröhlich KU, Sinner F, Tavernarakis N, Minois N,

Kroemer G, Madeo F. Induction of autophagy by spermidine promotes longevity. Nat Cell Biol. 2009; 11: 1305-1314.

46. Evason K, Collins JJ, Huang C, Hughes S, Kornfeld K. Valproic acid extends Caenorhabditis elegans lifespan. Aging Cell 2008; 7: 305-317.

47. Demidenko ZN, Shtutman M, Blagosklonny MV. Pharmacologic inhibition of MEK and PI-3K converges on the mTOR/S6 pathway to decelerate cellular senescence. Cell Cycle 2009; 8: 1896-1900.
